# Offshore platforms as novel ecosystems: A case study from Australia’s Northwest Shelf

**DOI:** 10.1002/ece3.8496

**Published:** 2022-02-12

**Authors:** Sean van Elden, Jessica J. Meeuwig, Richard J. Hobbs

**Affiliations:** ^1^ School of Biological Sciences The University of Western Australia Crawley WA Australia

**Keywords:** de facto MPAs, decommissioning, offshore platform ecology, oil and gas, stereo‐BRUVS

## Abstract

The decommissioning of offshore oil and gas platforms typically involves removing some or all of the associated infrastructure and the consequent destruction of the associated marine ecosystem that has developed over decades. There is increasing evidence of the important ecological role played by offshore platforms. Concepts such as novel ecosystems allow stakeholders to consider the ecological role played by each platform in the decommissioning process. This study focused on the Wandoo field in Northwest Australia as a case study for the application of the novel ecosystem concept to the decommissioning of offshore platforms. Stereo‐baited remote underwater video systems were used to assess the habitat composition and fish communities at Wandoo, as well as two control sites: a sandy one that resembled the Wandoo site pre‐installation, and one characterized by a natural reef as a control for natural hard substrate and vertical relief. We found denser macrobenthos habitat at the Wandoo site than at either of the control sites, which we attributed to the exclusion of seabed trawling around the Wandoo infrastructure. We also found that the demersal and pelagic taxonomic assemblages at Wandoo more closely resemble those at a natural reef than those which would likely have been present pre‐installation, but these assemblages are still unique in a regional context. The demersal assemblage is characterized by reef‐associated species with higher diversity than those at the sand control and natural reef control sites, with the pelagic community characterized by species associated with oil platforms in other regions. These findings suggest that a novel ecosystem has emerged in the Wandoo field. It is likely that many of the novel qualities of this ecosystem would be lost under decommissioning scenarios that involve partial or complete removal. This study provides an example for classifying offshore platforms as novel ecosystems.

## Graphical abstract

We assessed the marine communities at the Wandoo offshore oil platform in northwest Australia, and compared these communities with natural habitats. We found that the marine community at Wandoo more closely resembles that of a natural reef than the community that would have been present prior to the installation of the platform. The Wandoo platform can be classified as a novel ecosystem, which should be considered at the time of decommissioning.

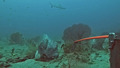

## 1 INTRODUCTION

Offshore oil and gas platforms (hereafter offshore platforms) have been a feature of continental shelf waters for over 70 years, with nearly 12,000 of these structures currently installed around the world (Aagard & Besse, 1973; Ars & Rios, 2017). When an offshore platform is no longer economically viable, a decision is made on the fate of the structure through a process referred to as decommissioning. In most cases, decommissioning involves complete removal of the platform from the marine environment for scrapping or recycling on land (Schroeder & Love, 2004). Complete removal is legislated as the default decommissioning method in many countries and regions, including Australia and the North Sea, as well as internationally under the United Nations Convention on the Law of the Sea (UNCLOS) and the 1996 Protocol to the London (Dumping) Convention (Chandler et al., 2017; Elizabeth, 1996; Techera & Chandler, 2015). However, the London Convention does permit *in situ* decommissioning for purposes other than disposal, and some regions have legislated such methods. In the Gulf of Mexico, platforms can be left either wholly or partially in place, or towed to a new location, under a program known as Rigs‐to‐Reefs (RTR, Reggio, 1987). Offshore platforms have been shown to form highly complex artificial reefs (Shinn, 1974), and RTR programs represent a method for preserving and maintaining these artificial reef communities that are established around offshore platforms over the decades they spend in the ocean, similar to the reefs formed by shipwrecks (Dauterive, 2000; Leewis et al., 2000). In situ decommissioning is a financially beneficial option for energy companies due to the excessive costs associated with complete removal (Dauterive, 2000), and this motivation is often used as an argument against rigs‐to‐reefs, particularly by environmental groups (Löfstedt & Renn, 1997).

Offshore platforms play various ecological roles, including acting as aggregation sites for marine megafauna (Haugen & Papastamatiou, 2019; Robinson et al., 2013), nurseries for juvenile fishes (Love et al., 2019; Nishimoto et al., 2019), and providing habitat for economically important and overfished species (Bond, Langlois, et al., 2018; Love et al., 2006). The presence of these offshore platforms creates new habitat, which can have a significant impact on fish production; platforms in California are some of the most productive fish habitats in the world, and platforms in Gabon have higher fish biomass than pristine reefs in the Pacific (Claisse et al., 2014; Friedlander et al., 2014). Fishing is excluded around offshore platforms in many countries, either by law as is the current case in Australia (Commonwealth of Australia, 2010), or by the presence of subsea infrastructure which can damage fishing equipment (de Groot, 1982). The partial or complete exclusion of fishing effectively creates de facto marine protected areas (MPAs) around offshore platforms (de Groot, 1982; Friedlander et al., 2014). The exclusion of fishing is particularly important in areas which are overfished, or where hard substrate is limited and infrastructure may be some of the only obstacles to trawling (de Groot, 1982; Fujii & Jamieson, 2016; Love et al., 2006; Schroeder & Love, 2002).

There is an increasing research focus around the world on the potential ecological importance of offshore platforms, and particularly on ensuring that the role of these platforms as ecosystems is considered in the decommissioning process (Bull & Love, 2019; Fowler et al., 2014, 2018; Macreadie et al., 2012; Meyer‐Gutbrod et al., 2020). An ecological perspective of offshore platforms allows scientists to apply restoration principles to the decommissioning process, in a similar way to terrestrial restoration of abandoned mine sites (Koch & Hobbs, 2007). The presence of offshore platforms modifies communities and habitats to such an extent that returning the site to its pre‐installation state may no longer be feasible or preferable (Sommer et al., 2019), and as such, the benefits of in situ decommissioning must be evaluated.

This assertion is congruent with the concept of novel ecosystems, which is intended to complement existing restoration practices. A novel ecosystem is one which has been altered by human activity and where restoration is not feasible or would result in the loss of ecosystem value (Hobbs et al., 2013). Recently, there have been attempts to apply restoration management concepts to offshore platforms in terms of: establishing ecological baselines for restoring the ecosystem post‐decommissioning (Fortune & Paterson, 2020); the potential for restoration paradigms to shift the discourse surrounding RTR decommissioning (Ounanian et al., 2019); and direct application of novel ecosystems criteria to offshore platforms (Schläppy & Hobbs, 2019; van Elden et al., 2019).

There is still a significant knowledge gap around the ecology of these platforms, particularly outside of the major northern hemisphere oil and gas producing regions. In Australia, only a limited number of studies exist on the fish and shark communities around offshore infrastructure (Bond, Langlois, et al., 2018; Bond, Partridge, et al., 2018; Fowler & Booth, 2012; McLean et al., 2019; Pradella et al., 2014; Thomson et al., 2021). Information on how ecological value is retained under varying decommissioning scenarios is needed at a time when the Australian government is reviewing legislation to potentially allow *in situ* decommissioning options (Offshore Resources Branch, 2018; Taylor, 2020). It is critical that we understand the ecological role platforms play in a regional context before the associated ecosystems are potentially lost due to decommissioning and restoration activity.

The offshore oil and gas producing region of northwest Australia, the Northwest Shelf (NWS), is comprised of over 40 production facilities and over 2000 km of subsea pipelines (Bond, Partridge, et al., 2018; Geoscience Australia, 2009). This is not a large number of platforms when compared with other locations around the world. However, the NWS is largely devoid of any significant natural hard substrate, and therefore offshore platforms contribute a significant portion of such habitat regionally, along with its associated fishes. This area was historically characterized by established macrobenthos communities made up of sponges, gorgonians, and soft corals on flat, sand inundated pavement (Evans et al., 2014). These macrobenthos communities were largely removed by pair‐trawling operations in the 1960s and 1970s (Fromont et al., 2016; Sainsbury et al., 1997). Previous studies on both platforms and pipelines on the NWS have found significant macrobenthos habitat associated with these structures, and abundance and richness of fish was higher on pipelines than on nearby natural habitats (Bond, Langlois, et al., 2018; Bond, Partridge, et al., 2018; McLean et al., 2018, 2019). These results suggest that the hard substrate provided by oil and gas infrastructure may modify the habitat and associated communities from their previously trawled state.

We investigate whether the presence of active offshore infrastructure at a site on the NWS has resulted in the emergence of a novel ecosystem, characterized by a shift in the structure of marine communities. Demersal and pelagic taxonomic assemblages, as well as macrobenthos communities, were documented around the infrastructure in the Wandoo oil field (Wandoo) over 3 years and six surveys and in contrast to two control sites: a sandy site (Control Sand) and a natural reef (Control Reef). Baseline (pre‐installation) ecological information for the Wandoo site was not collected, as has been the case for many older offshore platforms (Fortune & Paterson, 2020). As such, the Control Sand site acts as a proxy for the historical state of the Wandoo site. We determined historical state as the state of the environment immediately prior to the installation of the Wandoo infrastructure, and as such, this site would have been subject to trawling. Anthropogenic disturbance creates challenges in selecting historical baselines, and our baseline selection is congruent with the Anthropocene baseline concept (Kopf et al., 2015). The Control Reef site is characterized by a rocky substrate with significant physical relief, and similar in spatial extent to the infrastructure in the Wandoo field. Control Reef provides contrast to the Wandoo site in the form of a natural reef that is comparable in size (area) and depth (m). These two sites allowed us to both assess Wandoo as a novel ecosystem and predict how the marine communities would be altered under two different decommissioning scenarios. Specifically, complete removal may see the Wandoo site revert to a state more similar to the Control Sand site, and partial removal (topping) may lead to something more similar to the Control Reef site, due to the loss of the mid‐water hard substrate. We chose to use the post‐trawling state of the Northwest Shelf as our historical baseline, as if the Wandoo infrastructure were to be removed, this area would likely be exposed to trawling again. We used baited remote underwater video systems (BRUVS) to determine how taxonomic richness, abundance, biomass, fork length, and community assemblage structure varied between these sites, as well as intra‐ and inter‐annually. We hypothesize that the demersal and pelagic assemblages at Wandoo would more closely resemble those at the control reef site than those at the control sand site with respect to diversity, abundance, and size. We then evaluated our findings on the Wandoo field against the criteria for testing whether an offshore platform can be classified as a novel ecosystem (van Elden et al., 2019).

## 2 MATERIALS AND METHODS

### 2.1 Study sites

**FIGURE 1:**
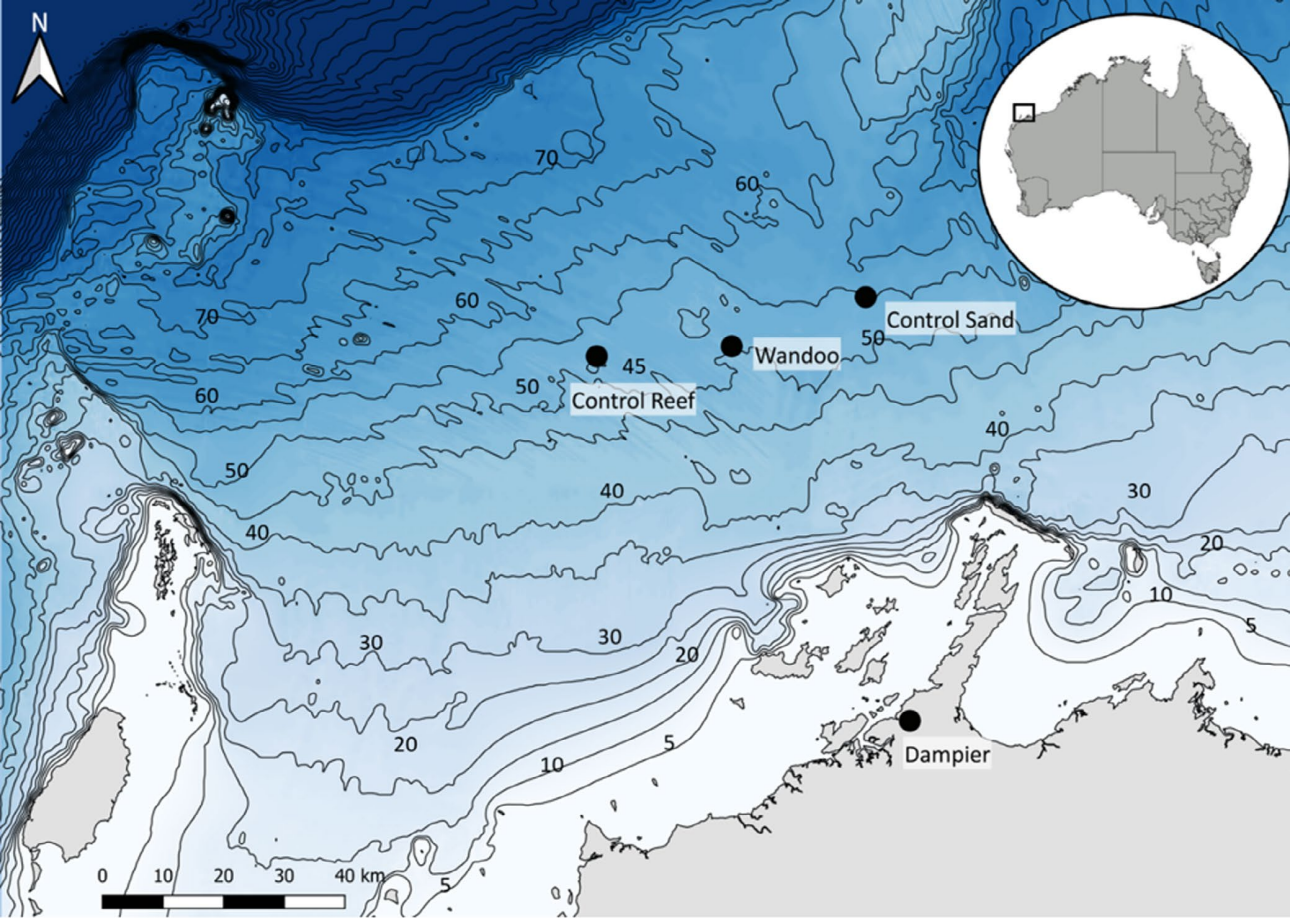
Location of the three study sites, Wandoo, Control Reef and Control Sand, approximately 75 km north‐west of Dampier, Western Australia

**FIGURE 2:**
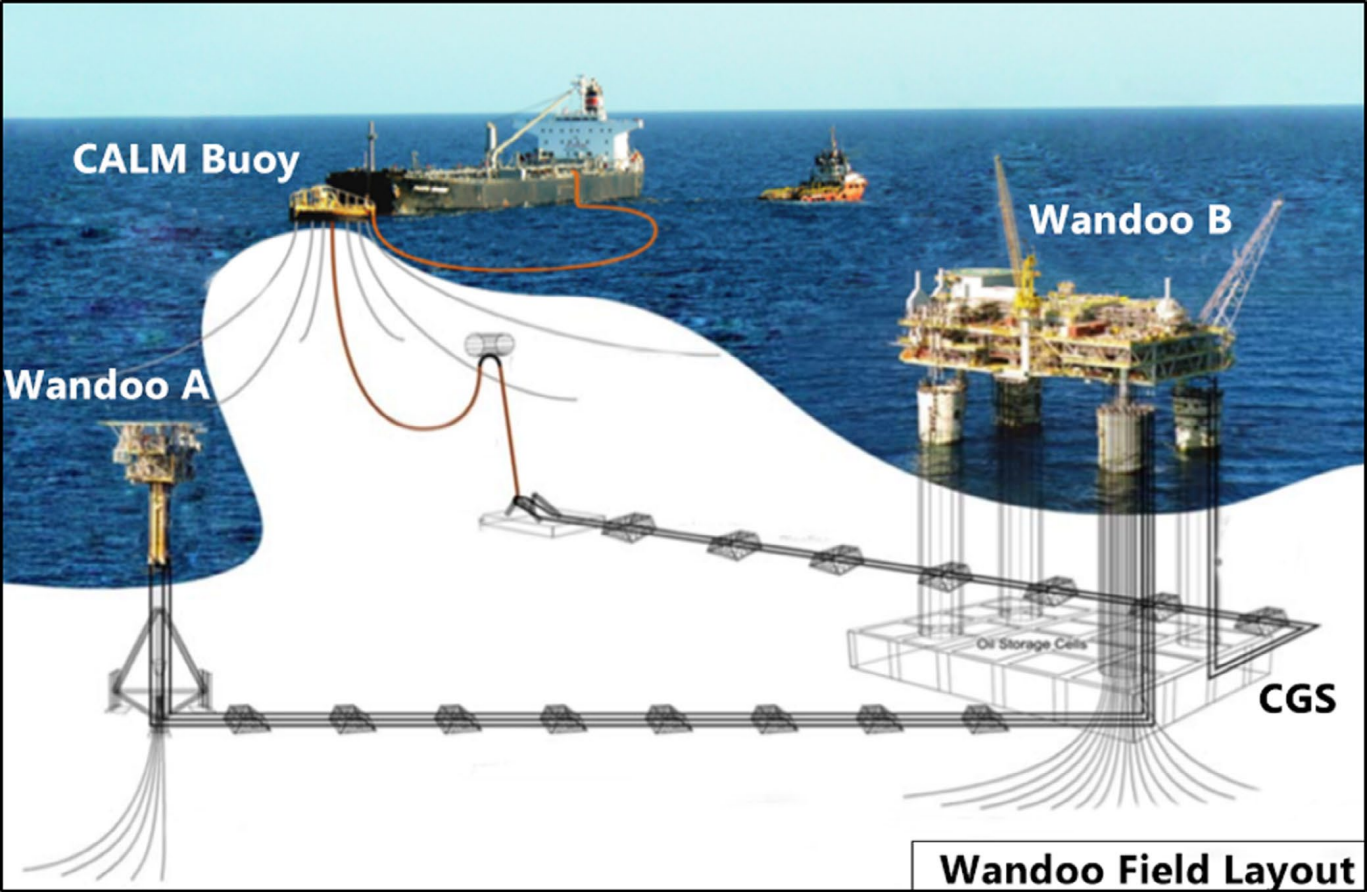
Wandoo oil field schematic adapted from Vermilion Oil and Gas Australia (2014). The infrastructure at the Wandoo field includes the unmanned monopod Wandoo A, the concrete gravity structure Wandoo B, the pipeline end manifold (PLEM), and the catenary anchored leg mooring (CALM) Buoy. Not to scale

The three sites sampled are located in the NWS region of northwest Australia, approximately 75 km northwest of Dampier, Western Australia (Figure 1). The sites are all situated in waters approximately 50–60 m deep. The Wandoo site (WN) is an active oil field leased by Vermilion Oil and Gas Australia Pty Ltd (Vermilion). This site contains oil production infrastructure including: Wandoo A, an unmanned monopod wellhead platform with a 2.5‐m‐diameter shaft supporting a helideck and production infrastructure; Wandoo B, a concrete gravity structure (CGS) made up of a 114 m long by 69 m wide caisson and four shafts, each 11 m in diameter, supporting the superstructure approximately 18 m above the sea surface; and a catenary anchored leg mooring (CALM) buoy, with six moorings and a Pipeline End Manifold (PLEM) below the buoy (Figure 2). The infrastructure at the Wandoo site is surrounded by a 500 m exclusion zone, within which only authorized vessels are permitted to operate (Commonwealth of Australia, 2010). These exclusion zones are in place around all offshore platforms in Australia, and represent some of the only areas on the Northwest Shelf fully protected from commercial fishing activity. Two control sites, comparable in depth to Wandoo, were also sampled: a flat sand‐dominated site, Control Sand (CS) comparable to the Wandoo site prior to infrastructure installation in 1994; and a reef site, Control Reef (CR) that is a natural structure comparable in dimension to the Wandoo infrastructure.

The CS site is situated approximately 15 km northeast of the Wandoo site (Figure 1) and is characterized by little to no physical relief and a dense, silty sand habitat. The CR site is located approximately 15 km west of the Wandoo site (Figure 1) and is characterized by a rocky reef, similar in spatial extent to the infrastructure in the Wandoo field, rising to approximately 20 m below the surface. Unlike the WN site, the CS and CR sites are accessible to commercial and recreational fishing.

### 2.2 Stereo‐baited underwater video systems

Stereo‐BRUVS are a non‐destructive, cost‐effective method for studying marine fauna (Cappo et al., 2006; Letessier et al., 2013; Letessier, Juhel, et al., 2015). They have been used to study abundance, biomass, diversity, distribution, and behavior in animals ranging from fish and sharks, to turtles, moray eels, and marine mammals (Barley et al., 2016; Letessier, Bouchet, et al., 2015; Spaet et al., 2016; Thompson et al., 2019; Whitmarsh et al., 2017). Seabed stereo‐BRUVS have been adapted to mid‐water environments, making them a useful tool for documenting highly mobile and elusive species (Bouchet et al., 2018; Letessier et al., 2013; Thompson et al., 2019). BRUVS‐derived data should be interpreted recognizing the potential impact of variable bait plumes (Whitmarsh et al., 2017), the potential higher representation of piscivores, and the relative nature of abundance estimates in contrast with density estimates generated by, for instance, underwater visual census (UVC, Langlois et al., 2010). Despite these constraints, BRUVS can be used to document clear signals in marine communities relative to other methods (Cappo et al., 2006; Lowry et al., 2012).

Seabed stereo‐BRUVS consist of two GoPro cameras mounted 80 cm apart on a horizontal base bar, each converging at an angle of four degrees to a common focal point. A galvanized steel mesh bait cage containing 800 g of crushed pilchards is attached to the end of a 1.5‐m‐long bait arm. Seabed stereo‐BRUVS are deployed at least 200 m apart for a minimum of 60 min.

Mid‐water stereo‐BRUVS consist of the same horizontal base bar as seabed stereo‐BRUVS, mounted on a 1.45‐m‐long steel upright to provide stability, and suspended 10 m below the surface. They are baited with 1 kg of crushed pilchards in a perforated bait canister on a 1.5‐m‐long bait arm, which acts as a rudder to keep the cameras facing down‐current for the duration of the deployment. Mid‐water stereo‐BRUVS are deployed for a minimum of 120 min, and in this study, are anchored to prevent entanglement with subsea infrastructure.

### 2.3 Data collection

Sampling was undertaken over 3 years, from 2017 to 2019, with twice‐yearly expeditions in the austral autumn and spring. Due to the significant tide range and variable weather conditions in the region, surveys were limited to a 10 day window over neap tides. In most of the surveys, it was only possible to sample two of the three study sites, and the three sites were therefore not sampled evenly between years and seasons. The WN site was sampled in both autumn and spring in all three years. The CR site was sampled in autumn and spring of 2017, autumn of 2018, and spring of 2019, while the CS site was sampled in autumn and spring of 2018 and autumn of 2019.

A total of 595 seabed stereo‐BRUVS and 530 mid‐water stereo‐BRUVS deployments were conducted over the 3‐year study period, using a random stratified sampling design. At the WN site, 14 sampling zones were established around the infrastructure, with seabed stereo‐BRUVS deployed in 10 zones around the structure, and mid‐water stereo‐BRUVS deployed 9 zones. All stereo‐BRUVS were deployed a minimum of 50 m away from any infrastructure at the Wandoo site so as to avoid collision and/or entanglement between the stereo‐BRUVS and the infrastructure. To ensure consistency in data collection, stereo‐BRUVS were deployed a minimum of 50 m away from the reef structure at the Control Reef site. All sampling was carried out during daylight hours to minimize the effect of crepuscular animal behavior. The sampling was conducted under UWA ethics permit RA/3/100/1484.

### 2.4 Data processing and treatment

Prior to each survey, individual stereo‐BRUVS were calibrated in an enclosed pool, according to standard protocols, using the CAL software (Harvey & Shortis, 1998; SeaGIS Pty Ltd, 2020). All video samples collected in the field were converted to AVI format using Xilisoft Video Converter Ultimate (Xilisoft Corporation, 2016) and videos were processed using the Eventmeasure software package (SeaGIS Pty Ltd, 2020). Processing commenced either once seabed stereo‐BRUVS had settled on the seabed, for a period of 60 min, or when the mid‐water stereo‐BRUVS had stabilized at 10 m depth following deployment, for a period of 120 min. All animals entering the field of view were identified to the lowest possible taxonomic level, and abundance was estimated using the conservative abundance metric MaxN, which is the maximum number of individuals of a given taxon in a single frame (Cappo et al., 2006). The appropriate length metric (e.g., fork length FL, disc width DW, or carapace length CL) was measured in stereo with individuals measured where they were well positioned relative to the camera and not occluded by other individuals. For seabed stereo‐BRUVS, the habitat visible in the field of view was broadly categorized into three groups: sand (bare substrate with no visible macrobenthos or other marine growth); sparse macrobenthos (predominantly bare substrate with <50% biotic cover); and dense macrobenthos (the visible substrate was dominated by more than 50% biotic cover).

For seabed stereo‐BRUVS, a sample was an individual rig deployment. For mid‐water stereo‐BRUVS, samples consisted of each set of five BRUVS deployed in a zone. This method mitigates the potential effect of highly mobile pelagic species being observed on multiple mid‐water deployments.

The video analysis yielded identification, abundance, and length data for each stereo‐BRUVS deployment. These data were analyzed as taxonomic richness (TR), total abundance (TA), and fork length (FL), respectively. Total biomass (TB) was calculated as the sum of mean weight of a given taxa on a given sample. Weight was calculated based on FL using taxon‐specific length weight relationships (LWR) sourced from Fishbase (Froese et al., 2019). Where the LWR was not available for a particular taxon, the LWR based on total length (TL) for that taxon was used, in combination with taxon‐specific TL:FL conversions. Where an animal was identified to genus or family, the Bayesian LWR was sourced from Fishbase (Froese et al., 2014). Taxon‐specific biomass estimates were calculated by multiplying the abundance of each taxon by the mean weight of that taxon. Marine mammals were excluded from the biomass estimates as they were multiple orders of magnitude heavier than the largest observed fish and heavily skewed the estimates. These four univariate metrics, TR, TA, TB, and FL, were analyzed separately for each survey in order to ensure like‐for‐like comparisons between sites. Annual and seasonal variability were also assessed for each site to determine the variability in the demersal and pelagic communities at each site over time. These analyses were also carried out at the level of survey, comparing annual variability separately for autumn and spring at each site, and seasonal variability (i.e., between spring and autumn) for each year at each site.

The prevalence of each taxon at each site was calculated by determining the percentage of seabed deployments or mid‐water zones on which the particular taxa were observed of the total for that site. The prevalence data were then used to determine the number of unique demersal and pelagic taxa for each site, by extracting taxa that were only recorded at one site. We did not count taxa which were recorded on only one mid‐water zone or seabed deployment per site, in order to eliminate chance sightings and possible incorrect identifications. Within the lists of unique taxa, any taxon that was only identified to genus or family was removed if there was a record from that genus or family at another site.

### 2.5 Statistical analyses

The categorized habitat data were analyzed using a Chi‐square contingency test to determine whether habitat varied significantly by site (Zar, 1999). Variation in the fish assemblage was tested using PERMANOVA as it is robust to data heterogeneity (Anderson, 2017). The linear variables of TA, TB, and FL were log_10_ transformed to stabilize variance (Zar, 1999). For each of these univariate measures, a Euclidean distance resemblance matrix was calculated and a PERMANOVA was applied based on unrestricted permutations (Anderson, 2017) with Site and Survey as fixed factors. Site was defined as Wandoo, Control Reef, or Control Sand, while Survey was defined as each of the six BRUVS surveys conducted in a particular season and year (e.g., Autumn 2017). Our main hypothesis was whether sites differed in their fish assemblages and the degree to which such differences varied temporally. To first determine whether sites differed, one‐way pairwise PERMANOVAs were applied within each survey period. We also similarly tested for differences between years and between seasons within sites. Repeated measures ANOVA was not used as the sampling through space and time varied randomly within the zones and seasons (Zar, 1999).

The assemblage composition data were treated differently from the univariate metrics. Species composition data were pooled across all surveys in preparation for the multivariate analyses for each sampling method. The data were analyzed by survey to ensure like‐for‐like comparisons between sites. Multivariate analyses were completed on the pelagic and demersal taxonomic assemblage data in terms of abundance and biomass to understand variations in species composition between sites as well as which variables explained this variation. We log(*x* + 1) transformed the assemblage data and calculated Bray–Curtis resemblance matrices for abundance and biomass of each species. Pairwise PERMANOVAs were applied to determine the differences between the demersal and pelagic species compositions of the three sites, across all surveys, in terms of both abundance and biomass. Canonical analysis of principal coordinates (CAP) was used in order to visualize a constrained ordination of the data on the basis of distance or dissimilarity.

A database of physical, chemical, and biological variables was also compiled in order to understand the potential environmental effects on taxonomic assemblages. Distances to marine features (e.g., coral reefs and seamounts) were calculated using bathymetry data following Yesson et al. (2021). Environmental data were derived from the following datasets:

- Geoscience Australia (GA) 250 m bathymetry (Whiteway, 2009);
- GA Australian submarine canyons (Huang et al., 2014);
- CSIRO Atlas of Regional Seas (CARS) (Ridgway et al., 2002); and
- Australia's Integrated Marine Observing System (IMOS) Moderate Resolution Imaging Spectroradiometer (MODIS) (IMOS, 2020)

A number of anthropogenic variables, such as time to market and distance from nearest human population, were also calculated based on human accessibility calculations undertaken by Maire et al. (2016). However, the three sites are almost exactly the same distance from the coast, so distance‐based variables were similar for all sites, and fishing effort data were not fine scale enough to separate the three sites. As such, the anthropogenic variables were excluded.

A Pearson's correlation was run to identify highly correlated independent variables with a correlation coefficient >0.6 (Havlicek & Peterson, 1976). Analyses included only one of any highly correlated variables in a given test. A distance‐based linear model (DistLM) was used to determine the relationship between these variables and the assemblage data across all surveys. All analyses were completed using the Primer 7 software package with the PERMANOVA + add‐on (Anderson et al., 2015).

## 3 RESULTS

In the six surveys across 3 years, we counted 35,070 individuals from 358 taxa, representing 85 families (Appendices 4 and 5). The total biomass of these animals was 42.5 tons, excluding marine mammals. Of the 358 taxa, 252 (70%) were unique to the demersal samples, 44 (13%) were unique to the pelagic samples, and 62 (17%) of the taxa were common to both sets of samples. Fork length of demersal taxa ranged from a 2 cm unidentified juvenile to a 260.4 cm wedgefish *Rhynchobatus* sp. Three families accounted for 57% of all demersal animals recorded: jacks (Carangidae; 32%), threadfin breams (Nemipteridae; 14%), and damselfishes (Pomacentridae; 11%), while the most prevalent demersal species was the starry triggerfish *Abalistes stellatus*, occurring on 91% of deployments. Pelagic taxa ranged in fork length from a 0.86 cm juvenile leatherjacket Monacanthidae sp., to a 6.27 m northern minke whale *Balaenoptera acutorostrata*, with the largest fish being a 3.93 m tiger shark *Galeocerdo cuvier*. Two families accounted for 79% of all pelagic animals recorded: herrings (Clupeidae; 40%) and jacks (Carangidae; 39%). The most prevalent pelagic taxon was scads *Decapterus* sp., occurring on 72% of deployments. Threatened species included two Critically Endangered taxa, wedgefishes *Rhynchobatus* sp. and great hammerhead *Sphyrna mokarran*, and two Endangered species, dusky shark *Carcharhinus obscurus* and zebra shark *Stegostoma tigrinum* (Dudgeon et al., 2019; Rigby et al., 2019a, 2019b).

### 3.1 Environment

**FIGURE 3:**
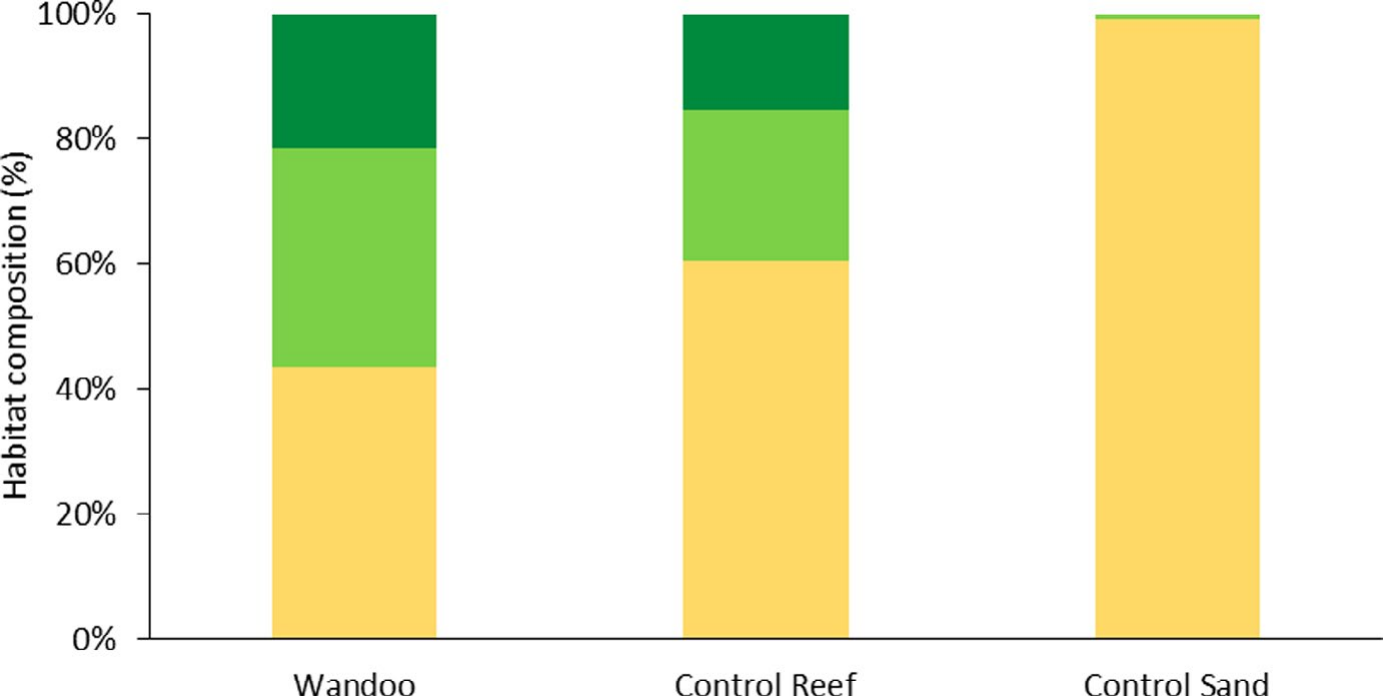
Percentage habitat composition for each of the three sites. The habitat types are sand (yellow), sparse macrobenthos (light green) and dense macrobenthos (dark green)

Observed habitats across the three sites, included sand, and macrobenthos which consisted of sponges, sea whips, crinoids, soft corals, and gorgonians. Macrobenthos coverage was both sparse (<50%) and dense (>50%). Habitat differed significantly across the three sites with the WN site characterized by a higher percentage of samples dominated by dense and sparse macrobenthos relative to the other two sites (*X^2^*_(2,_ *_N_*_=417)_ = 91.1, *p* < .001). Macrobenthos was present on 57% of the deployments at WN, with sand dominating deployments at CR and CS (60% and 99%, respectively; Figure 3). The highest percentage of dense macrobenthos also occurred at WN (22%), compared with 15% at CR and none at CS.

There was limited environmental variability between the sites (Appendix 1). As expected, based on sampling design, depth was not significantly different between WN and CR (*t*_468_ = 1.87, *p* = .06), although CS was significantly but only marginally deeper than WN and CR (*t*_418_ = 16.8, *p* < .001 and *t*_298_ = 7.87, *p* < .001, respectively). Mean sea surface temperature (SST) in autumn was similar at WN and CR (*t*_218_ = 1.18, *p* = .26), but was approximately one degree higher at CS than at WN and CR (*t*_218_ = 6.15, *p* < .001 and *t*_148_ = 6.19, *p* < .001, respectively; Appendix 1).

Mean SST in spring did not differ significantly between WN and CS (*t*_198_ = 1.30, *p* = .21), but was significantly higher at CR than at WN and CS (*t*_248_ = 2.08, *p* = .038 and *t*_148_ = 2.75, *p* = .007, respectively). Mean chlorophyll concentration (Chl‐a) in autumn was higher at WN than CR and CS (*t*_218_ = 3.34, *p* = .003 and *t*_218_ = 2.62, *p* = .002, respectively), with no difference between the latter two sites (*t*_148_ = 0.39, *p* = .71). In spring, mean Chl‐a was significantly higher at CR than both WN and CS (*t*_248_ = 7.84, *p* < .001 and *t*_148_ = 4.55, *p* < .001, respectively), with no significant difference between WN and CS (*t*_198_ = 1.21, *p* = .22; Appendix 1).

### 3.2 Demersal richness, abundance, biomass, and fork length

**FIGURE 4:**
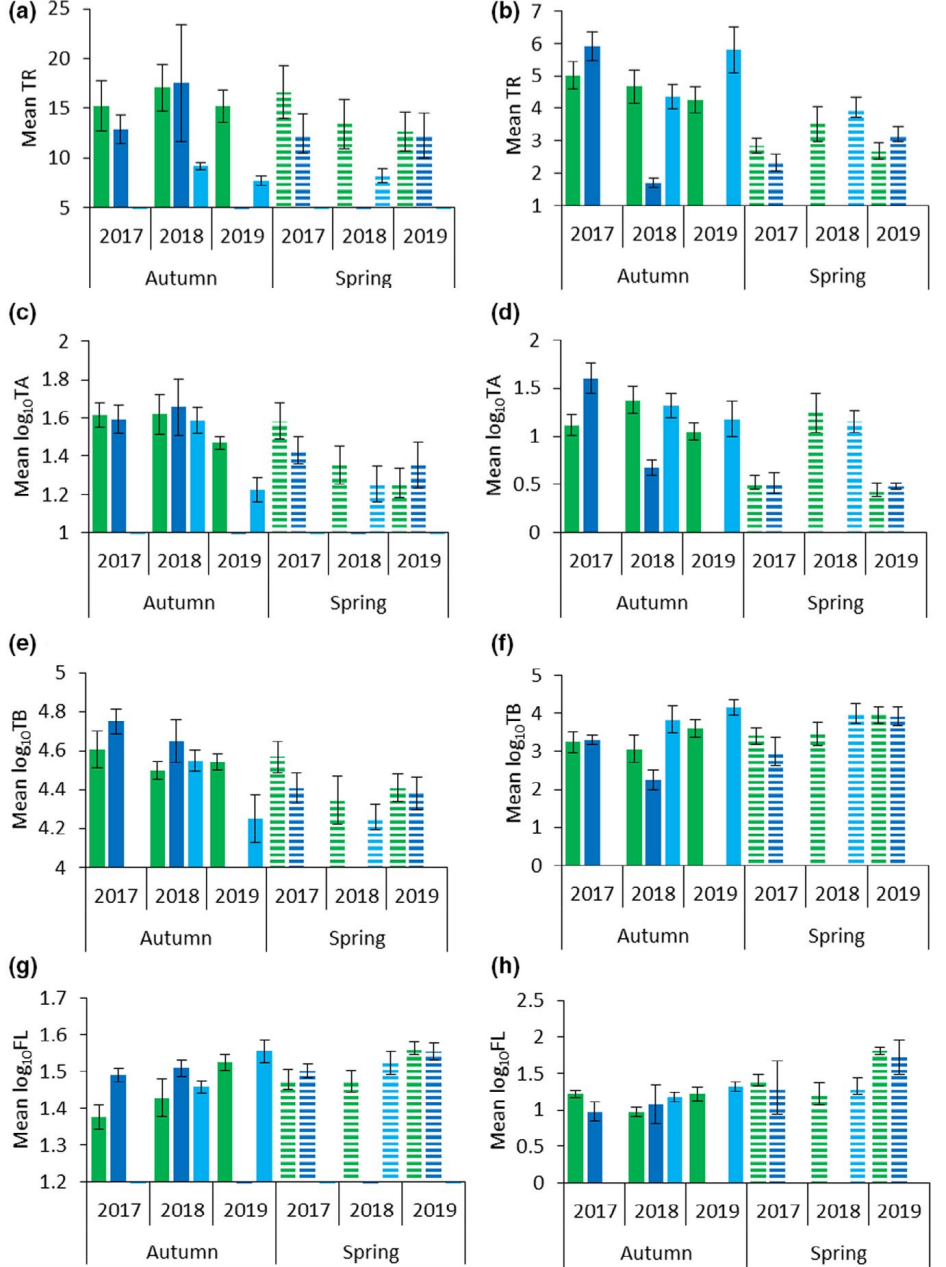
Mean values with standard errors (SE) for taxonomic richness (TR), and logged values of total abundance (TA), total biomass (TB) fork length (FL) by survey for demersal (left) and pelagic (right) communities at the three sites: Wandoo (green); Control Reef (dark blue) and Control Sand (light blue). Solid bars indicate autumn surveys while broken bars indicate spring surveys

The mean demersal richness was 13.1 ± 0.90 SE and ranged between 7.7 and 17.5 taxa per sample. There was significant variation in richness between sites in four of the six surveys, where richness was higher at WN than at the control site sampled in the same survey (Figure 4a; Table 1). The only seasonal variation was in 2018 at WN, when richness was higher in autumn than spring, and the only annual variation was at CS, where richness was higher in 2018 than 2019 (Appendix 2).

**TABLE 1:** Pairwise PERMANOVA tests comparing demersal and pelagic variation between sites for each survey, for taxonomic richness (TR), log total abundance (log_10_TA), log total biomass (log_10_TB), and log fork length (log_10_FL)

Survey × Site	Groups	df	TR		log_10_TA		log_10_TB		log_10_FL	
			*t*	*p* (perms)	*t*	*p* (perms)	*t*	*p* (perms)	*t*	*p* (perms)
Demersal										
Autumn 2017	Control Reef, Wandoo	58	1.94	.059 (146)	0.88	.393 (997)	1.91	.063 (999)	3.29	*.004 (996)
Autumn 2018	Control Reef, Control Sand	59	4.24	*.001 (138)	0.97	.341 (996)	1.37	.175 (997)	2.17	*.031 (997)
Autumn 2018	Control Reef, Wandoo	48	0.13	.885 (168)	0.07	.936 (994)	1.86	.063 (995)	3.39	*.002 (997)
Autumn 2018	Control Sand, Wandoo	71	6.99	*.001 (142)	1.31	.176 (998)	0.34	.721 (997)	2.27	*.024 (996)
Autumn 2019	Control Sand, Wandoo	62	5.82	*.001 (131)	5.04	*.001 (999)	2.78	*.004 (998)	1.42	.173 (999)
Spring 2017	Control Reef, Wandoo	71	2.14	*.029 (162)	1.80	.082 (997)	1.61	.114 (996)	0.81	.432 (998)
Spring 2018	Control Sand, Wandoo	66	4.09	*.001 (132)	1.07	.298 (998)	1.21	.226 (998)	1.09	.287 (996)
Spring 2019	Control Reef, Wandoo	76	0.33	.742 (169)	1.76	.079 (996)	0.07	.939 (997)	0.65	.544 (998)
Pelagic										
Autumn 2017	Control Reef, Wandoo	16	1.43	.194 (39)	2.59	*.026 (981)	0.20	.839 (976)	3.14	*.014 (978)
Autumn 2018	Control Reef, Control Sand	12	4.93	*.003 (123)	3.65	*.005 (805)	3.05	*.016 (779)	1.04	.314 (762)
Autumn 2018	Control Reef, Wandoo	12	4.10	*.003 (375)	3.47	*.009 (768)	1.55	.159 (775)	0.94	.39 (775)
Autumn 2018	Control Sand, Wandoo	16	0.48	.65 (121)	0.31	.764 (981)	1.52	.154 (975)	2.21	*.032 (977)
Autumn 2019	Control Sand, Wandoo	9	2.06	.089 (68)	0.74	.511 (312)	1.54	.152 (318)	0.75	.462 (315)
Spring 2017	Control Reef, Wandoo	16	1.44	.182 (213)	0.03	.975 (972)	0.92	.37 (974)	0.54	.608 (976)
Spring 2018	Control Sand, Wandoo	16	0.84	.419 (128)	0.38	.697 (974)	1.33	.198 (974)	0.58	.578 (976)
Spring 2019	Control Reef, Wandoo	16	1.49	.179 (151)	0.40	.704 (910)	0.14	.88 (981)	0.83	.395 (984)

Note

Degrees of freedom (df) are reported. *p*‐values in bold and with an asterisk are <.05, and the number of permutations (perms) are reported in parentheses.

Abundance ranged from 17.9 to 77.4 individuals per sample, with a mean of 43.5 ± 4.99 SE. Abundance was consistent between sites in most surveys, only differing in Autumn 2019 when abundance at WN was higher than at CS (Figure 4b; Table 1). In terms of annual variability at WN, abundance in autumn was higher in both 2017 and 2018 than in 2019. In spring, abundance was also higher in 2017 than both 2018 and 2019. There was more annual variability in abundance at WN than at CR or CS, and seasonal abundance followed the same pattern at all sites, with abundance generally being higher in autumn than spring (Appendix 2).

Mean biomass was 44.5 kg ± 3.31 SE, and ranged from 28.2 kg to 70.2 kg per sample. Similar to abundance, biomass was consistent between sites for all surveys except Autumn 2019, when biomass was higher at WN than CS (Figure 4c; Table 1). The only annual variation was at CS, where biomass was higher in 2018 than 2019. Biomass was consistent between seasons at WN but was higher in autumn than spring at the control sites (Appendix 2).

Fork length ranged from 24.8 cm to 38.5 cm per sample, with a mean of 32.6 cm ± 1.05 SE. Fork length was consistent between sites in most surveys, but was higher at WN in Autumn 2017 and Autumn 2018 (Figure 4d; Table 1). Fork length was generally higher in spring than autumn, and higher in 2019 at WN and CS (Appendix 2).

### 3.3 Pelagic richness, abundance, biomass, and fork length

Mean pelagic richness was 3.9 ± 0.15 SE, with a range of 1.3 to 8.4 taxa per zone. Richness in the Autumn 2018 survey was significantly higher at WN and CS than at CR, but was consistent between sites in all other surveys (Figure 4e; Table 1). Annual and seasonal richness was consistent in most surveys at both WN and CS, but there was significant annual and seasonal variation at CR (Appendix 3).

Abundance ranged from 1.3 to 271 individuals per zone, with a mean of 29.6 ± 4.7 SE. Abundance was consistent between sites in four of the six surveys, and was higher at WN than at CR in the other two surveys (Figure 4f; Table 1). Annual variability occurred at WN in spring, but at the control sites in autumn, and there was seasonal variability in abundance at WN and CS (Appendix 3).

Mean biomass was 48.5 kg ± 5.7 SE and ranged from 7.5 g to 429 kg per zone. Biomass was significantly lower at CR than CS in Autumn 2018, but was consistent between sites across all other surveys (Figure 4g; Table 1). The only annual or seasonal variation in biomass was at CR, where biomass was higher in Autumn 2017 than Autumn 2018 (Appendix 3).

Fork length ranged from 3.8 cm to 182 cm per zone, with a mean of 37.5 cm ± 3.3 SE. Fork length was higher in Autumn 2017 at WN than CR, and higher in Autumn 2018 at CS than WN. Fork length was consistent between sites in all other surveys (Figure 4h; Table 2). There was no annual or seasonal variability in fork length at the control sites, with annual variability in three of the six surveys at WN (Appendix 3).

**TABLE 2:** Pairwise PERMANOVA results comparing abundance and biomass of the pelagic and demersal taxonomic assemblages among sites: Wandoo (WN); Control Sand (CS); and Control Reef (CR). Degrees of freedom (df) are reported. *p*‐values in bold and with an asterisk are < .05, and the number of permutations (perms) are reported in parentheses

	Abundance			Biomass		
	Groups	*t* (df)	*p* (perms)	Groups	*t* (df)	*p* (perm)
Demersal	CR, CS	4.46 (218)	*.001 (998)	CR, CS	4.85 (218)	*.001 (999)
	CR, WN	3.48 (329)	*.001 (996)	CR, WN	3.53 (329)	*.001 (998)
	CS, WN	6.76 (317)	*.001 (999)	CS, WN	7.53 (317)	*.001 (997)
Pelagic	CR, CS	1.86 (52)	*.002 (999)	CR, CS	1.63 (52)	*.013 (998)
	CR, WN	1.88 (82)	*.001 (998)	CR, WN	2.20 (82)	*.001 (999)
	CS, WN	2.30 (72)	*.001 (999)	CS, WN	2.44 (72)	*.001 (999)

### 3.4 Community assemblages

**FIGURE 5:**
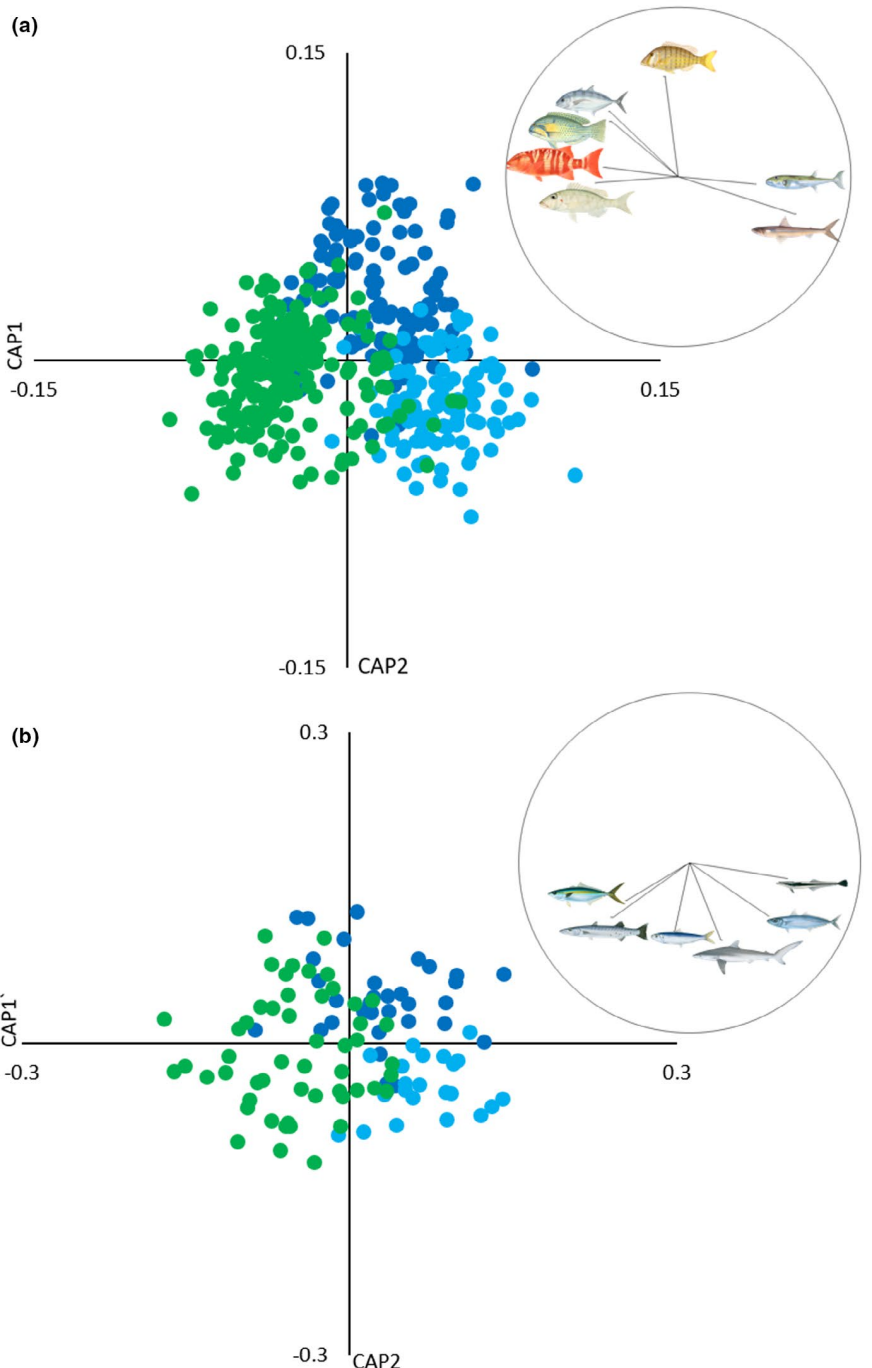
Canonical analysis of principal coordinates (CAP) for abundance of (a) demersal and (b) pelagic taxonomic assemblages at Wandoo (green); Control Reef (dark blue) and Control Sand (light blue). Species clockwise from top in (a) are: bluespotted emperor *Lethrinus punctulatus*, northwest blowfish *Lagocephalus sceleratus*, brushtooth lizardfish *Saurida undosquamis*, galloper *Symphorus nematophorus*, spot‐cheek emperor *Lethrinus rubrioperculatus*, bluespotted tuskfish *Choerodon cauteroma*, and turrum *Carangoides fulvoguttatus*. Taxa clockwise from top in (b) are: live sharksucker *Echeneis naucrates*, scads *Decapterus* sp., silky shark *Carcharhinus falciformis*, herrings *Clupeidae* sp., great barracuda *Sphyraena barracuda*, and rainbow runner *Elegatis bipinnulata*. Images © R. Swainston/anima.fish

**FIGURE 6:**
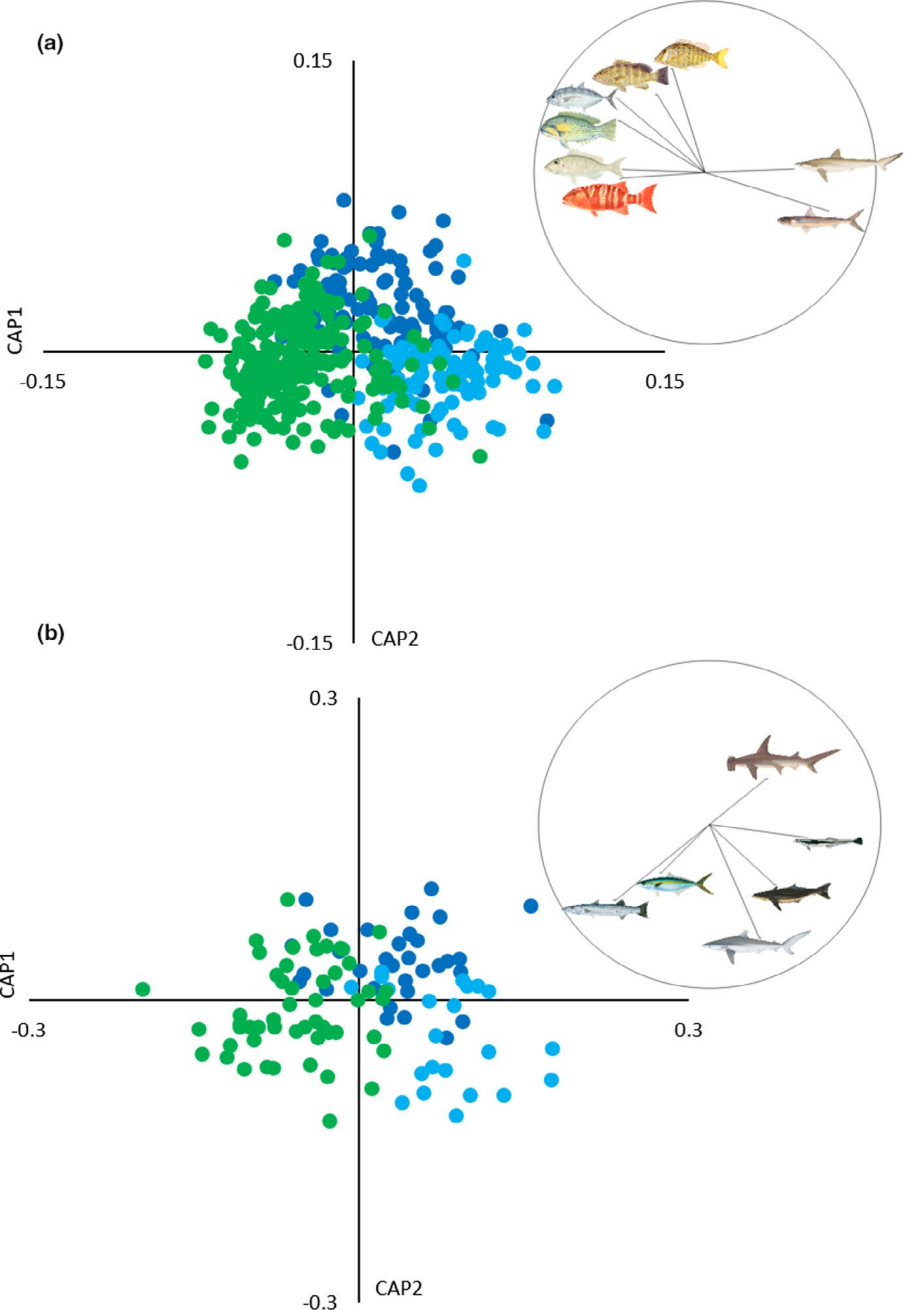
Canonical analysis of principal coordinates (CAP) for biomass of (a) demersal and (b) pelagic taxonomic assemblages at Wandoo (green); Control Reef (dark blue) and Control Sand (light blue). Species clockwise from top in (a) are: bluespotted emperor *Lethrinus punctulatus*, milk shark *Rhizoprionodon acutus*, brushtooth lizardfish *Saurida undosquamis*, galloper *Symphorus nematophorus*, spot‐cheek emperor *Lethrinus rubrioperculatus*, bluespotted tuskfish *Choerodon cauteroma*, turrum *Carangoides fulvoguttatus*, and areolate grouper *Epinephelus areolatus*. Taxa clockwise from top in (b) are: great hammerhead *Sphyrna mokarran*, live sharksucker *Echeneis naucrates*, cobia *Rachycentron canadum*, silky shark *Carcharhinus falciformis*, rainbow runner *Elegatis bipinnulata*, and great barracuda *Sphyraena barracuda*. Images © R. Swainston/anima.fish

There was strong separation of both demersal and pelagic taxonomic assemblages between sites, with abundance and biomass at each site characterized by unique species assemblages. Demersal and pelagic taxonomic assemblages were significantly different from each other at all sites, in terms of both abundance (Figure 5) and biomass (Figure 6) (Table 2). The DistLM analysis showed that the three environmental variables, depth, SST, and Chl‐a, did not explain a sufficient proportion of the variance in the assemblage data, and as such, these analyses were excluded.

Demersal abundance (Figure 5a) and biomass (Figure 6a) at WN were driven by reef‐associated species, namely galloper *Symphorus nematophorus* and spot‐cheek emperor *Lethrinus rubrioperculatus*. Both species usually occurred in low abundance but were prevalent across deployments at WN (38% and 40%, respectively; Appendix 4). Abundance and biomass at CR were driven by different reef‐associated species than at WN, namely bluespotted emperor *Lethrinus punctulatus* (the name most commonly used for this unresolved species; Moore et al., 2020) and turrum *Carangoides fulvoguttatus*, both of which occurred in large schools, while biomass was also driven by areolate grouper *Epinephelus areolatus*, a more solitary species. There was some overlap in taxonomic assemblages between WN and CR, driven by bluespotted tuskfish *Choerodon cauteroma*. Abundance at CS was characterized by northwest blowfish *Lagocephalus sceleratus*, a species associated with offshore reefs and sandy habitats, and brushtooth lizardfish *Saurida undosquamis*, a sand or mud bottom–associated species. These species occurred in relatively low numbers but were highly prevalent on deployments at this site (51% and 80%, respectively; Appendix 4). Brushtooth lizardfish also characterized biomass at CS, along with the milk shark *Rhizoprionodon acutus*, also associated with sandy habitats. Habitat associations were sourced from Fishbase (Froese et al., 2019).

There were 17 demersal taxa from 11 families observed only at WN, compared with five unique taxa from five families at CR and four taxa from four families at CS (Table 3). Many of the demersal species unique to WN are reef‐associated species, and WN was the only site where unidentified larval‐stage juvenile fishes were present. Two demersal species recorded only at WN were observed on over 10% of deployments, namely the pickhandle barracuda *Sphyraena jello*, and giant sea catfish *Netuma thalassina* (Appendix 4).

**TABLE 3:** Abundance, biomass, and prevalence of taxa observed at a single site at WN (16 demersal and 1 pelagic species), CR (5 demersal and 0 pelagic species), and CS (4 demersal and 0 pelagic species) based on demersal and pelagic sampling records. Species marked with an asterisk are commonly caught commercially and/or recreationally in the North Coast Bioregion (Rome & Newman, 2010).

	Family	Binomial	Common names	Abundance	Biomass (g)	Prevalence (%)
Demersal						
Wandoo	Apogonidae	*Apogonidae* sp.	Cardinalfishes	41	37.65	1.4
	Ariidae	*Netuma thalassina*	Giant sea catfish	2	4696.20	12.1
	Blenniidae	*Meiacanthus* sp.	Combtooth blennies	2	22.33	0.9
	Carangidae	*Carangoides dinema*	Shadow trevally	2	1435.95	0.9
	Carangidae	*Carangoides orthogrammus*	Island trevally	1	1308.26	2.3
	Carangidae	*Caranx sexfasciatus*	Bigeye trevally	4	12604.88	1.4
	Carangidae	Caranx tille*	Tille trevally	1	4334.75	0.9
	Ginglymostomatidae	*Nebrius ferrugineus*	Tawny nurse shark	1	5064.06	2.3
	Juvenile	*Juvenile* sp.	Unidentified juvenile	1	0.05	0.9
	Lethrinidae	*Gymnocranius euanus*	Paddletail seabream	1	554.72	1.4
	Pinguipedidae	*Parapercis* sp.	Grubfishes	1	18.09	2.3
	Pomacentridae	*Pomacentrus nagasakiensis*	Blue‐scribbled damsel	3	6.68	0.9
	Serranidae	Cephalopholis sonnerati*	Tomato rockcod	2	978.65	0.9
	Serranidae	Epinephelus chlorostigma*	Brownspotted grouper	1	1024.97	0.9
	Serranidae	Epinephelus malabaricus*	Malabar grouper	1	4097.69	1.9
	Sphyraenidae	*Sphyraena jello*	Pickhandle barracuda	2	7637.25	10.7
Control Reef	Chaetodontidae	*Chaetodon auriga*	Threadfin butterflyfish	2	445.78	1.7
	Labridae	*Bodianus bilunulatus*	Saddleback pigfish	1	439.87	2.6
	Lethrinidae	Lethrinus atkinsoni*	Yellowtail emperor	2	1265.86	2.6
	Monacanthidae	*Eubalichthys caeruleoguttatus*	Bluespotted leatherjacket	1	822.73	1.7
	Muraenidae	*Gymnothorax undulatus*	Undulated moray	1	0.97	1.7
Control Sand	Carangidae	Seriola rivoliana*	Highfin amberjack	11	3280.25	1.9
	Clupeidae	*Clupeidae* sp.	Herrings	334	22240.67	1.9
	Congridae	*Gorgasia* sp.	Garden eels	31	2525.02	2.9
	Lutjanidae	Pristipomoides multidens*	Goldband snapper	1	3498.20	1.9
Pelagic						
Wandoo	Carangidae	*Elagatis bipinnulata*	Rainbow runner	22	112861.56	15.0

Pelagic assemblages followed similar patterns in terms of abundance (Figure 5b) and biomass (Figure 6b) to those observed in the demersal assemblages. Abundance and biomass at WN were driven by great barracuda *Spyhraena barracuda* and rainbow runner *Elegatis bipinnulata*. Great barracuda were usually solitary, but frequently observed at WN (60% of zones, Appendix 5), while rainbow runner was observed less frequently (15% of zones) but in large schools. There was some overlap in abundance between WN and CS, characterized by herrings (Clupeidae spp.), which were observed on 25% of zones at WN and 41% at CS. Abundance and biomass at CS was driven by silky sharks *Carcharhinus falciformis* and live sharksuckers *Echeneis naucrates*, and biomass was also characterized by cobia *Rachycentron canadum*. Abundance at CR was not strongly characterized by any particular species, while biomass was driven by great hammerheads *Sphyrna mokarran*, which was always solitary and only observed on 16% of zones. WN was the only site where any unique pelagic taxa were recorded, with rainbow runner not observed at either of the control sites (Table 3).

## 4 DISCUSSION

The demersal and pelagic community assemblages in the Wandoo field are distinct from those that would have existed prior to the installation of the infrastructure. The habitat around Wandoo is dominated by macrobenthos, in contrast to the sand‐dominated habitat that would have likely prevailed historically (Sainsbury et al., 1993). As a result, the Wandoo demersal assemblage is characterized by reef‐associated rather than sand‐associated taxa. The pelagic assemblage at Wandoo is different from the other two sites, driven by species associated with offshore platforms on the NWS as well as in other regions around the world (Friedlander et al., 2014; McLean et al., 2019; Reynolds et al., 2018). Overall, the demersal and pelagic assemblages more closely resemble a natural reef than the assemblages that would have existed pre‐installation, which is congruent with our hypothesis. However, the composition of these assemblages is still unique to Wandoo, suggesting the emergence of a novel ecosystem.

While the focus of our study was on the fish assemblages, we saw clear differences in the habitat at the three sites. The proliferation of macrobenthos at the otherwise flat WN site, in contrast to the barren sand habitat at CS, likely reflects the exclusion of seabed trawling at WN. The WN site also had higher demersal fish richness than the control sites in most surveys which suggests that habitat composition is a driver of diversity in these demersal communities, as has been found elsewhere on the NWS (Anon, 2019). The Pilbara Offshore mesoscale region, within which the study sites are located, is a biodiversity hotspot for sponges (Fromont et al., 2016). However, as much of the macrobenthos biomass was removed by seabed trawling (Sainsbury et al., 1993), most of the habitat in this region has been simplified. The impact of trawling is clear at CS and the area surrounding the reef at CR, with both sites dominated by bare sand. In contrast, WN excludes seabed trawling up to 500 m from the infrastructure, and exhibited similar macrobenthos communities to other oil and gas infrastructure on the NWS (Bond, Langlois, et al., 2018; McLean et al., 2019).

The demersal community at WN was more diverse and reef associated than the communities at the control sites. The higher demersal richness at WN is congruent with studies from Brazil, the Persian Gulf, and Gabon, which describe offshore platforms as diversity hotspots (Fonseca et al., 2017; Friedlander et al., 2014; Torquato et al., 2017). High diversity is often associated with structural complexity of hard substrate (Friedlander & Parrish, 1998), and this association was observed in ROV surveys of the Wandoo infrastructure (Tothill, 2019). This study sampled areas around the infrastructure with little to no hard substrate, suggesting a large area of influence or “ecological halo” around the Wandoo infrastructure. The species that characterized the demersal taxonomic assemblage at WN, namely galloper and spot‐cheek emperor, are both valued as fishing species: galloper is a prized sport fish, while spot‐cheek emperor is a food fish targeted by recreational and commercial fishers (Anon, 2019; Rome & Newman, 2010). These species occupy different habitats, with galloper inhabiting coral reefs and spot‐cheek emperor inhabiting sand/rubble areas (2019a). Spot‐cheek emperor was rarely observed at either control site, despite the habitat at CR arguably being more suitable than that found at WN. Fishing activity, which is excluded at WN, may be the reason for the lower prevalence of this species at the control sites.

The similarity in pelagic communities across sites in terms of all four metrics was expected, given the three sites are located relatively close to each other and the highly mobile nature of pelagic species. For example, great barracuda have been shown to travel 12 km in a day and can migrate over 100 km, while silky sharks can travel up to 60 km a day (Bonfil, 2008; O’Toole et al., 2011). While these species are highly mobile, there was still strong distinction in the taxonomic assemblages between the three sites. The two species which characterized the taxonomic assemblage at WN, great barracuda and rainbow runner, are often associated with offshore platforms. Great barracuda is a commonly recorded species around offshore platforms in the Gulf of Mexico (Reynolds et al., 2018; Wetz et al., 2020), accounting for 33.2% of the biomass at offshore platforms in Gabon (Friedlander et al., 2014), and was recorded in 100% of remotely operated vehicle (ROV) transects at another platform on the NWS (McLean et al., 2019). Rainbow runner have also been recorded around platforms in the Gulf of Mexico, Gabon, and Brunei (Chou et al., 1992; Friedlander et al., 2014; Reynolds et al., 2018). Great hammerheads characterized biomass at CR, which was attributed to the fact that these are large animals and would have a significant effect on biomass even if present in low numbers, especially as there was not a particularly high abundance of any other species at this site. The pelagic taxonomic assemblage at CS was characterized by silky sharks, which were observed within minutes of the vessel's arrival to conduct surveys at this site. This behavior and the associated high abundance and biomass of this species were attributed to the frequent commercial fishing activity that occurs at this site. There are commercial line, trap, and trawl fisheries operating throughout this area, including CS and, to a lesser extent, CR (WAFIC, 2020). This population of silky sharks is thought to be opportunistically targeting the discards from the commercial fishing vessels as a food source, which would explain their high abundance at a site otherwise scarce in the typical prey of this species, which includes scombrids, carangids, snappers, and groupers (Compagno, 1984).

A distinct marine community exists at WN with various taxa not observed at natural habitats. Many of the 17 unique demersal species at WN are reef associated, but species such as paddletail seabream *Gymnocranius euanus* and blue‐scribbled damsel *Pomacentrus nagasakiensis* are found in sandy areas adjacent to reefs (2019a). This suggests that the combination of sand and macrobenthos habitats around WN, itself a de facto artificial reef, is a key component of the high diversity and unique assemblage at this site. Reef‐associated species tend to have strong site fidelity and post‐settlement ranges of less than 50 m (Frederick, 1997). While it is possible that some species recruit to WN from natural sites, and certainly would have done when the platform was first installed, the high number of species unique to WN suggests that fish are being produced at the platform, rather than simply being attracted from natural habitats. Tothill (2019) observed juvenile fishes in the mid‐water (10–22 m) sections of Wandoo, providing further evidence of fish production. There was only one pelagic species unique to a single site, which may reflect the relatively mobile nature of pelagic animals. Rainbow runner were only observed at the WN site, which could be attributed to the association of this species with offshore platforms around the world (Chou et al., 1992; Reynolds et al., 2018). Offshore platforms can function as fish aggregation devices (FADs), aggregating fish by facilitating foraging and school formation (Dagorn et al., 2000; Haugen & Papastamatiou, 2019). Rainbow runner are thought to primarily aggregate around FADs to prey on small FAD‐associated pelagic fishes (Xuefang et al., 2013), and it is possible that the vertical hard structure at WN is providing enhanced foraging opportunity for this species.

The exclusion of fishing around WN has created a de facto MPA, as has been reported at other offshore platforms (Friedlander et al., 2014; Fujii & Jamieson, 2016; Love et al., 2006). Seabed trawling on the NWS in the 1970s not only removed much of the macrobenthos habitat but also resulted in a significant shift in fish composition (Sainsbury et al., 1993). The trawl catch shifted from being dominated by emperors (*Lethrinus* sp.) and snappers (*Lutjanus* sp.) to being dominated by lizardfish (*Saurida* sp.) and threadfin bream (*Nemipterus* sp.), with the abundance of lizardfishes greater by an order of magnitude (Sainsbury et al., 1993; Thresher et al., 1986). This relationship between habitat and species composition was also observed in this study: macrobenthos habitat was present at WN and CR, both of which were characterized by emperors. In contrast, at CS, the habitat was almost completely devoid of macrobenthos, and the species composition was characterized by brushtooth lizardfish. Lizardfishes feed on benthic fishes, particularly on juveniles of other species, and are estimated to collectively consume 4 × 10^7^ fishes per day on the NWS (Thresher et al., 1986). Demersal communities dominated by lizardfish, such as CS, would therefore have been significantly impacted by the proliferation of this genus. The de facto MPA has also resulted in a large ecological halo around the WN infrastructure. The ecological halo around offshore platforms and artificial reefs is usually around 15–34 m, with abundance and diversity similar to natural habitats beyond this distance (Reeds et al., 2018; Scarcella et al., 2011; Stanley & Wilson, 1996). In contrast, diversity at WN was higher than natural habitats at more than 50 m from the infrastructure. It is likely that the larger ecological halo at WN is due to the 500 m exclusion zone which was not present around the infrastructure in other ecological halo studies. The WN ecological halo is driven by recovery of macrobenthos habitat due to the exclusion of trawling activity.

### 4.1 Wandoo as a novel ecosystem

The ecosystem in the Wandoo field clearly has novel attributes when compared with natural systems in the region; however, this assertion is not, on its own, sufficient to warrant labelling Wandoo a novel ecosystem. Van Elden et al. (2019) used the novel ecosystems definition developed by Hobbs et al. (2013) to establish three criteria for evaluating offshore platforms as novel ecosystems:

1. The abiotic, biotic, and social components of the system differ from those that prevailed historically. The addition of hard substrate through the installation of the Wandoo infrastructure altered the abiotic component of the system. It is impossible to quantify the historical baseline of the biotic component, however, the findings of this study show that the biotic components of the Wandoo ecosystem, in terms of habitat and marine communities, are distinct from those found at a proxy of their pre‐installation (post‐trawling) historical state, i.e., the Control Sand site. The major social driver of this ecosystem is the exclusion of fishing activity, which has been detrimental to large areas of the NWS. The de facto MPA effect of Wandoo has been particularly important in providing a refuge for fishes and allowing macrobenthos communities to recover.
2. The ecosystems have a tendency to self‐organize and manifest novel qualities without intensive human management. The Wandoo ecosystem, like those found at most other offshore platforms, is an unintended consequence of the installation of the platform and therefore is not subject to any human management. The only management undertaken is cleaning of sections of the subsea structure, but this activity only removes a small portion of the marine growth. The factors that allow this ecosystem to thrive, such as the exclusion of fishing and the provision of hard substrate, are artefacts of the presence of the platform.
3. Novel ecosystems are prevented from returning to their historical states by practical limitations, in the form of ecological, environmental, and social considerations. Wandoo is due to remain operational for at least a further 10 years, which is a significant social consideration as the presence of the infrastructure is central to this ecosystem. When Wandoo is decommissioned, it is possible that complete removal will allow the ecosystem to return to its pre‐installation state due to exposure to trawling, but the evidence presented here on the unique ecology of Wandoo should provide an ecological consideration against complete removal, thereby preventing a return to the historical state of the site.

Based on these criteria, Wandoo may be classified as a novel ecosystem. The environment and ecology of the site have been altered, a self‐organizing ecosystem with novel qualities has emerged, and the presence of the platform prevents the ecosystem from returning to its post‐trawling state.

### 4.2 Implications for decommissioning

We have used proxies for different decommissioning scenarios, which can provide a broad idea of how the Wandoo ecosystem might look post‐decommissioning. We suggest that the Control Sand site is a proxy for complete removal, as this site is already a proxy for the Wandoo site without infrastructure. If the Wandoo infrastructure was completely removed, there would be a significant loss in diversity, particularly in terms of reef‐associated species. Pelagic species associated with mid‐water structure, such as great barracuda and rainbow runner, are also likely to no longer be present at this site. Commercial and recreational fishing activity would likely recommence in the field post‐decommissioning, as the petroleum safety zone would no longer be in effect and there would be no significant hard structure to prevent seabed trawling.

Topping, a second decommissioning scenario, would result in partial removal of Wandoo down to around 25 m below the surface. This method has been applied to shallow‐water platforms in the United States (Ajemian et al., 2015). The reef at the Control Reef site rises to around 30 m below the surface, making this a close approximation to a topped Wandoo. This scenario would also result in the loss of pelagic species associated with structure, but would result in the retention of more of the demersal community than complete removal. There would be some losses: the shallower portions of Wandoo are important for juveniles, exhibit higher richness and abundance than deeper portions, and are characterized by small reef fish such as damselfishes (Tothill, 2019). Indeed larval‐stage juveniles were absent from the Control Reef site, and abundance of small demersal species such as damselfishes was generally lower than at the Wandoo site. It is likely that even under a topping scenario there would no longer be any exclusion of fishing activity around the remaining part of the platform. Seabed trawling could still occur in the areas surrounding the infrastructure that were previously protected by the petroleum safety zone.

Partial or complete removal of the Wandoo platform will likely have adverse impacts on a number of taxa and alter the role of the infrastructure as a novel ecosystem, specifically in terms of the artificial reef and associated ecological halo. Partial removal would be less detrimental in that it would also still afford protection to the macrobenthos from seabed trawling. However, there is significant ecological benefit in retaining the mid‐water sections of the infrastructure, for both pelagic species and juvenile reef‐associated species, and leaving the platform standing in place would maintain these benefits. Additional aspects that should also be considered include the role of the infrastructure for seabirds, marine megafauna, and macrobenthos communities attached to the infrastructure, as have been reported from other offshore platforms around the world (Bond, Partridge, et al., 2018; Ronconi et al., 2015; Thomson et al., 2021; Todd et al., 2019). The exclusion of fishing is a critical component of the large ecological halo present at Wandoo; however, the petroleum safety zone would likely cease to exist post‐decommissioning. We would recommend that post‐decommissioning protection from fishing, in the form of a no‐take MPA, should be considered.

The installation of infrastructure in the Wandoo field has resulted in the emergence of a novel ecosystem with distinct ecological characteristics not found at natural sites in the region. The demersal and pelagic communities more closely resemble reef communities than those present pre‐installation, but are still unique from those found at natural habitats in the region. The novel ecosystem at Wandoo also acts as a refuge for these communities, functioning as a de facto MPA in a region impacted by historical and current fishing activity. This MPA not only protects fish communities but also has allowed the macrobenthos to recover from the impacts of seabed trawling. Many of the novel characteristics of the Wandoo ecosystem would be lost under decommissioning scenarios that involve partial or complete removal, and the impact of decommissioning on fauna such as seabirds is still unknown. Recognizing the Wandoo field as a novel ecosystem provides a mechanism for recognizing the ecological role played by the Wandoo infrastructure, and underlines the need to consider the ecological role of each offshore platform prior to decommissioning.

## CONFLICT OF INTEREST

This project is funded by Vermilion Oil and Gas Australia (VOGA). VOGA has 100% operating interest in the Wandoo field where this work was conducted. VOGA did not participate in the design of the study, analysis of the data, or development of the manuscript.

## AUTHOR CONTRIBUTIONS

**Sean van Elden:** Conceptualization (lead); Data curation (lead); Formal analysis (lead); Investigation (lead); Writing – original draft (lead); Writing – review & editing (equal). **Jessica J. Meeuwig:** Conceptualization (supporting); Data curation (supporting); Formal analysis (supporting); Investigation (supporting); Writing – original draft (supporting); Writing – review & editing (equal). **Richard J. Hobbs:** Conceptualization (supporting); Data curation (supporting); Formal analysis (supporting); Investigation (supporting); Writing – original draft (supporting); Writing – review & editing (equal).

## Data Availability

Survey data can be accessed through Dryad (https://doi.org/10.5061/dryad.mpg4f4r1s).

## APPENDIX 1

Environmental data for each survey used in the DistLM analyses, including start and end dates, depth, sea surface temperature (SST), and chlorophyll concentration (Chl‐a). Data were derived from: Geoscience Australia 250 m bathymetry (Whiteway, 2009) and Australia's Integrated Marine Observing System (IMOS) Moderate Resolution Imaging Spectroradiometer (MODIS) (IMOS, 2020). The sites are as follows: Wandoo (WN); Control Reef (CR); and Control Sand (CS)

**Table:**

Survey	Start Date	End Date	Site	Depth (m)			SST (°C)			Chl‐a (mg/m^3^)		
				Min	Max	Mean ± SD	Min	Max	Mean ± SD	Min	Max	Mean ± SD
Autumn 2017	4/05/2017	9/05/2017	CR	48.1	57.6	53.2 ± 2.02	27.8	27.9	27.9 ± 0.03	0.42	0.47	0.46 ± 0.02
			WN	48.9	55.2	52.4 ± 1.64	27.9	28.0	28 ± 0.02	0.36	0.49	0.4 ± 0.04
Autumn 2018	19/04/2018	26/04/2018	CR	47.3	56.7	53 ± 2.5	30.5	30.6	30.6 ± 0.07	0.15	0.17	0.17 ± 0.01
			CS	53.1	56.7	54.8 ± 0.78	30.5	30.7	30.6 ± 0.1	0.16	0.19	0.19 ± 0.01
			WN	50.3	55.7	52.5 ± 1.39	30.4	30.6	30.5 ± 0.07	0.17	0.19	0.19 ± 0.01
Autumn 2019	25/04/2019	30/04/2019	CS	53.0	55.0	54.1 ± 0.63	28.6	28.6	28.7 ± 0.01	0.69	0.79	0.75 ± 0.04
			WN	50.0	55.0	52.9 ± 1.31	28.4	28.6	28.5 ± 0.04	0.75	0.96	0.89 ± 0.06
Spring 2017	28/09/2017	4/10/2017	CR	43.1	56.6	52.7 ± 2.99	24.9	25.0	25 ± 0.04	0.61	0.66	0.64 ± 0.02
			WN	49.3	57.5	52.4 ± 1.39	24.8	24.9	24.9 ± 0.02	0.38	0.42	0.41 ± 0.01
Spring 2018	3/09/2018	19/09/2018	CS	53.3	57.2	55.3 ± 1.19	23.7	23.8	23.8 ± 0.02	0.30	0.31	0.31 ± 0.01
			WN	50.2	56.7	52.7 ± 1.5	23.5	23.5	23.6 ± 0.02	0.21	0.23	0.23 ± 0.01
Spring 2019	7/09/2019	11/09/2019	CR	43.0	56.0	52.6 ± 2.99	23.2	23.3	23.3 ± 0.03	0.23	0.25	0.24 ± 0.01
			WN	50.0	54.0	52.3 ± 1.17	23.2	23.3	23.3 ± 0.03	0.23	0.34	0.26 ± 0.04

## APPENDIX 2

Pairwise PERMANOVA comparing demersal variation between years for autumn and spring at each site for taxonomic richness (TR), log total abundance (log_10_TA), log total biomass (log_10_TB), and log fork length (log_10_FL). Degrees of freedom (df) are reported. *p*‐values in bold and with an asterisk are <.05, and the number of permutations (perms) are reported in parentheses

**Table:**

	Groups	df	TR		log_10_TA		log_10_TB		log_10_FL	
			*t*	*p* (perms)	*t*	*p* (perms)	*t*	*p* (perms)	*t*	*p* (perms)
Wandoo										
Autumn	2017, 2018	63	0.502	.601 (161)	0.256	.805 (999)	1.479	.133 (995)	0.145	.875 (992)
	2017, 2019	71	0.679	.496 (150)	3.725	***.001** (997)	1.01	.307 (999)	4.908	***.001** (997)
	2018, 2019	68	1.24	.226 (147)	3.312	***.004** (997)	0.859	.394 (999)	4.685	***.001** (998)
Spring	2017, 2018	66	1.335	.179 (159)	2.268	***.020** (996)	1.706	.089 (995)	0.053	.958 (998)
	2017, 2019	78	2.014	.058 (190)	3.53	***.001** (997)	1.462	.124 (998)	2.687	***.009** (997)
	2018, 2019	72	0.633	.538 (161)	1.206	.211 (996)	0.367	.719 (997)	3.07	***.004** (995)
Seasonal	2017	69	0.216	.837 (168)	0.792	.436 (996)	0.568	.578 (995)	2.204	***.021** (997)
	2018	60	2.13	***.041** (82)	3.72	***.002** (995)	1.255	.218 (995)	2.356	***.019** (997)
	2019	80	1.86	.053 (163)	3.009	***.002** (997)	1.718	.112 (998)	1.265	.222 (998)
Control Reef										
Autumn	2017, 2018	43	1.498	.149 (160)	0.256	.805 (999)	1.262	.224 (996)	0.547	.631 (999)
Spring	2017, 2019	69	0.449	.672 (151)	0.123	.891 (998)	0.154	.876 (996)	2.256	***.024** (997)
Seasonal	2017	60	0.343	.767 (131)	2.44	***.012** (997)	4.344	***.001** (998)	0.236	.825 (996)
Control Sand										
Autumn	2018, 2019	65	2.58	***.013** (50)	3.118	***.003** (996)	2.054	***.035** (997)	4.392	***.001** (997)
Seasonal	2018	77	0.947	.34 (74)	3.72	***.001** (995)	3.058	***.003** (992)	2.143	***.034** (997)

## APPENDIX 3

Pairwise permanova comparing pelagic variation between years for autumn and spring at each site, in terms of taxonomic richness (TR), log total abundance (log_10_TA), log total biomass (log_10_TB), and log fork length (log_10_FL. Degrees of freedom (df) are reported. *p*‐values in bold and with an asterisk are <.05, and the number of permutations (perms) are reported in parentheses

**Table:**

	Groups	df	TR		log_10_TA		log_10_TB		log_10_FL	
			*t*	*p* (perms)	*t*	*p* (perms)	*t*	*p* (perms)	*t*	*p* (perms)
Wandoo										
Autumn	2017, 2018	16	0.518	.615 (130)	1.476	.161 (978)	0.38079	.689 (982)	2.983	***.007** (977)
	2017, 2019	14	1.257	.234 (62)	0.452	.661 (952)	0.96021	.349 (958)	0.028	.98 (951)
	2018, 2019	14	0.592	.618 (212)	1.843	.092 (943)	1.1664	.281 (962)	2.14	.063 (952)
Spring	2017, 2018	16	1.167	.265 (218)	3.397	***.003** (973)	0.13979	.892 (974)	1.147	.256 (975)
	2017, 2019	16	0.452	.677 (144)	0.741	.462 (957)	1.8217	.1 (972)	4.45	***.001** (973)
	2018, 2019	16	1.398	.179 (125)	3.718	***.001** (966**)**	1.3543	.192 (971)	3.831	***.003** (967)
Seasonal	2017	16	4.49	***.002** (262)	4.621	***.001** (975)	0.45032	.651 (980)	2.064	.059 (973)
	2018	16	1.534	.149 (275)	0.548	.583 (981)	0.81364	.455 (980)	1.507	.181 (987)
	2019	14	3.376	.008 (146)	5.363	***.001** (942)	1.0773	.252 (963)	5.734	***.001** (955)
Control Reef										
Autumn	2017, 2018	12	6.83	***.001** (170)	4.212	***.002** (787)	4.0458	***.001** (804)	1.016	.319 (802)
Spring	2017, 2019	16	2.483	***.030** (230)	0.319	.742 (961)	2.081	.052 (980)	2.054	.053 (975)
Seasonal	2017	16	6.932	***.001** (393)	5.712	***.001** (977)	0.75851	.462 (983)	1.729	.09 (978)
Control Sand										
Autumn	2018, 2019	11	1.959	.066 (83)	0.627	.528 (554)	0.58776	.591 (533)	1.303	.226 (543)
Seasonal	2018	16	0.663	.525 (64)	0.993	.339 (980)	0.34781	.722 (983)	1.123	.279 (973)

## APPENDIX 4

Prevalence (%) of demersal species recorded at WN, CR, and CS. Prevalence refers to the number of deployments on which a taxon was observed, out of the total number of deployments at that site

**Table:**

Binomial	WN	CR	CS
*Abalistes stellatus*	92.1	95.7	84.6
*Acanthocybium solandri*	0.5	–	–
*Acanthurus auranticavus*	2.8	6.0	–
*Acanthurus blochii*	0.9	–	–
*Acanthurus grammoptilus*	1.9	0.9	–
*Acanthurus* sp.	3.3	6.0	–
*Acanthurus xanthopterus*	–	0.9	–
*Aetobatus ocellatus*	–	0.9	–
*Aipysurus laevis*	0.9	5.2	1.0
*Aipysurus* sp.	0.9	0.9	1.0
*Alectis ciliaris*	0.9	0.9	–
*Alectis indica*	0.5	–	–
*Alepes vari*	1.4	3.4	–
*Aluterus monoceros*	–	–	1.0
*Aluterus scriptus*	0.5	–	3.8
*Amblyeleotris* sp.	0.5	–	–
*Amblygobius phalaena*	0.5	–	–
*Apogonidae* sp.	1.4	–	–
*Apolemichthys trimaculatus*	–	0.9	–
*Aprion virescens*	0.5	–	1.0
*Argyrops bleekeri*	0.9	–	–
*Argyrops spinifer*	37.7	24.1	5.8
*Arothron* sp.	–	0.9	–
*Arothron stellatus*	0.5	–	–
*Aspidontus dussumieri*	1.9	1.7	–
*Aspidontus taeniatus*	–	0.9	–
*Asteroidea* sp.	0.9	5.2	1.0
*Asteroidea* sp.	–	0.9	–
*Atule mate*	1.4	0.9	1.0
*Balistidae* sp.	0.5	1.7	–
*Blenniidae* sp.	0.9	0.9	–
*Bodianus bilunulatus*	–	2.6	–
*Bodianus perditio*	10.2	16.4	–
*Bodianus solatus*	0.5	2.6	–
*Bodianus* sp.	0.9	–	–
*Bothus pantherinus*	0.5	–	–
*Bothus* sp.	2.8	–	4.8
*Brachyura* sp.	0.5	–	1.9
*Caesionidae* sp.	0.5	–	–
*Canthigaster* sp.	0.5	–	–
*Carangidae* sp.	8.4	12.1	27.9
*Carangoides armatus*	–	–	1.0
*Carangoides chrysophrys*	32.6	13.8	13.5
*Carangoides coeruleopinnatus*	69.8	43.1	17.3
*Carangoides dinema*	0.9	–	–
*Carangoides fulvoguttatus*	58.6	57.8	4.8
*Carangoides gymnostethus*	47.4	61.2	50.0
*Carangoides oblongus*	0.5	–	–
*Carangoides orthogrammus*	2.3	–	–
*Carangoides* sp.	7.4	3.4	15.4
*Caranx ignobilis*	2.8	2.6	2.9
*Caranx melampygus*	0.5	–	–
*Caranx papuensis*	1.4	2.6	–
*Caranx sexfasciatus*	1.4	–	–
*Caranx* sp.	0.9	–	–
*Caranx tille*	0.9	–	–
*Carcharhinidae* sp.	0.5	–	–
*Carcharhinus amblyrhynchos*	5.1	1.7	–
*Carcharhinus amboinensis*	1.4	–	1.0
*Carcharhinus falciformis*	–	–	1.0
*Carcharhinus leucas*	0.5	0.9	–
*Carcharhinus limbatus*	0.5	0.9	–
*Carcharhinus melanopterus*	0.5	–	–
*Carcharhinus obscurus*	–	–	1.9
*Carcharhinus plumbeus*	1.4	0.9	3.8
*Carcharhinus sorrah*	–	–	1.0
*Carcharhinus* sp.	2.8	6.0	10.6
*Caretta caretta*	–	0.9	–
*Cephalopholis boenak*	0.5	–	–
*Cephalopholis sonnerati*	0.9	–	–
*Cephalopholis* sp.	1.9	–	1.0
*Chaetodon auriga*	–	1.7	–
*Chaetodon lineolatus*	–	0.9	–
*Chaetodontidae* sp.	–	0.9	–
*Chaetodontoplus duboulayi*	23.7	15.5	1.0
*Chaetodontoplus personifer*	24.2	15.5	1.0
*Chaetodontoplus* sp.	–	1.7	–
*Chelmon marginalis*	0.5	0.9	–
*Chiloscyllium punctatum*	1.4	0.9	–
*Chlorurus* sp.	0.5	–	–
*Choerodon cauteroma*	47.0	44.8	1.9
*Choerodon schoenleinii*	0.5	1.7	–
*Choerodon vitta*	–	0.9	–
*Chromis fumea*	21.4	11.2	–
*Chromis* sp.	0.5	0.9	1.0
*Chromis westaustralis*	0.5	–	–
*Chrysiptera tricincta*	13.0	3.4	–
*Cirrhibarbis* sp.	0.9	0.9	–
*Cirrhilabrus* sp.	3.3	1.7	–
*Cirrhitidae* sp.	0.5	–	–
*Clupeidae* sp.	–	–	1.9
*Congrogadus* sp.	–	–	1.0
*Coradion altivelis*	0.5	0.9	–
*Coradion chrysozonus*	0.9	2.6	–
*Coradion* sp.	0.5	–	–
*Coris caudimacula*	16.7	2.6	2.9
*Coris pictoides*	2.8	–	–
*Coris* sp.	–	0.9	–
*Crinoidea* sp.	0.9	8.6	1.0
*Cromileptes altivelis*	2.3	0.9	–
*Dasyatidae* sp.	4.2	0.9	2.9
*Decapterus* sp.	2.3	1.7	8.7
*Diagramma labiosum*	18.1	7.8	1.0
*Diploprion bifasciatum*	2.3	5.2	–
*Dischistodus perspicillatus*	–	0.9	–
*Echeneis naucrates*	32.1	38.8	49.0
*Echinoidea* sp.	–	0.9	2.9
*Elapidae* sp.	6.0	2.6	1.9
*Epinephelus areolatus*	26.0	29.3	–
*Epinephelus bilobatus*	24.7	20.7	–
*Epinephelus chlorostigma*	0.9	–	–
*Epinephelus coioides*	1.9	1.7	–
*Epinephelus lanceolatus*	0.5	–	–
*Epinephelus malabaricus*	1.9	–	–
*Epinephelus multinotatus*	11.2	21.6	1.9
*Epinephelus polyphekadion*	0.5	0.9	–
*Epinephelus* sp.	7.0	8.6	1.0
*Eubalichthys caeruleoguttatus*	–	1.7	–
*Eviota* sp.	0.5	–	–
*Feroxodon multistriatus*	0.9	3.4	8.7
*Fistularia commersonii*	0.9	2.6	–
*Fistularia* sp.	1.9	0.9	–
*Galeocerdo cuvier*	0.9	0.9	1.0
*Gastropoda* sp.	0.9	–	–
*Glaucostegus typus*	0.5	–	–
*Gnathanodon speciosus*	27.0	16.4	2.9
*Gobiidae* sp.	3.7	11.2	1.9
*Gorgasia* sp.	–	–	2.9
*Gymnocranius euanus*	1.4	–	–
*Gymnocranius grandoculis*	13.5	5.2	–
*Gymnocranius griseus*	18.1	6.9	1.0
*Gymnocranius microdon*	3.3	–	–
*Gymnocranius* sp.	2.8	2.6	–
*Gymnothorax flavimarginatus*	0.5	–	–
*Gymnothorax javanicus*	0.5	–	–
*Gymnothorax mccoskeri*	–	0.9	–
*Gymnothorax* sp.	1.4	1.7	–
*Gymnothorax thyrsoideus*	0.5	–	–
*Gymnothorax undulatus*	–	1.7	–
*Haemulidae* sp.	1.4	–	–
*Hemigaleidae* sp.	–	–	1.0
*Hemigymnus melapterus*	–	0.9	–
*Heniochus acuminatus*	1.4	2.6	–
*Heniochus diphreutes*	0.9	0.9	–
*Heniochus* sp.	0.5	0.9	–
*Heteroconger hassi*	–	–	1.0
*Heteroconger* sp.	–	–	1.0
*Hydrophis major*	0.5	–	–
*Hydrophis ocellatus*	–	1.7	–
*Hydrophis* sp.	4.2	0.9	–
*Hydrozoa* sp.	0.5	0.9	–
*Iniistius pavo*	0.5	–	3.8
*Juvenile* sp.	0.9	–	–
*Labridae* sp.	10.2	1.7	1.0
*Labroides dimidiatus*	14.0	5.2	1.0
*Lagocephalus lunaris*	0.5	7.8	10.6
*Lagocephalus sceleratus*	12.6	32.8	51.0
*Lagocephalus* sp.	0.5	–	4.8
*Leptojulis cyanopleura*	3.3	0.9	–
*Lethrinidae* sp.	0.5	2.6	–
*Lethrinus amboinensis*	–	0.9	–
*Lethrinus atkinsoni*	–	2.6	–
*Lethrinus erythropterus*	–	0.9	–
*Lethrinus laticaudis*	0.5	–	–
*Lethrinus miniatus*	9.3	11.2	–
*Lethrinus nebulosus*	25.6	4.3	–
*Lethrinus olivaceus*	17.2	10.3	–
*Lethrinus punctulatus*	30.2	44.0	1.9
*Lethrinus rubrioperculatus*	40.5	12.1	–
*Lethrinus* sp.	2.8	3.4	–
*Lutjanus argentimaculatus*	1.4	1.7	–
*Lutjanus carponotatus*	0.5	–	–
*Lutjanus erythropterus*	10.2	6.0	–
*Lutjanus johnii*	–	0.9	–
*Lutjanus lemniscatus*	1.9	4.3	–
*Lutjanus malabaricus*	0.9	0.9	1.9
*Lutjanus monostigma*	0.5	–	–
*Lutjanus russellii*	–	0.9	–
*Lutjanus sebae*	41.4	31.0	3.8
*Lutjanus* sp.	1.9	6.0	1.0
*Lutjanus vitta*	2.3	1.7	1.0
*Megalaspis cordyla*	0.9	0.9	–
*Meiacanthus* sp.	0.9	–	–
*Microdesmidae* sp.	7.4	3.4	1.0
*Monacanthidae* sp.	0.5	1.7	1.9
*Mullidae* sp.	7.4	0.9	1.9
*Mulloidichthys flavolineatus*	0.5	–	2.9
*Muraenidae* sp.	0.5	–	–
*Naso brevirostris*	–	0.9	–
*Naso fageni*	0.5	–	–
*Naso* sp.	0.5	3.4	1.0
*Natator depressa*	0.5	–	–
*Nebrius ferrugineus*	2.3	–	–
*Nemipteridae* sp.	0.5	6.0	1.0
*Nemipterus furcosus*	38.1	52.6	67.3
*Nemipterus* sp.	17.2	18.1	33.7
*Nemipterus sp1*	7.0	0.9	21.2
*Neotrygon* sp.	0.5	–	–
*Netuma thalassina*	12.1	–	–
*Octopus* sp.	0.5	0.9	–
*Ophichthidae* sp.	–	0.9	–
*Ophiuroidea* sp.	0.5	–	2.9
*Octopoda* sp.	0.5	0.9	–
*Teuthida* sp.	–	0.9	–
*Ostraciidae* sp.	–	0.9	–
*Oxycheilinus orientalis*	0.5	–	–
*Paguridae* sp.	7.9	11.2	20.2
*Palinuridae* sp.	0.5	–	–
*Parachaetodon ocellatus*	1.9	0.9	–
*Paracirrhites* sp.	0.5	–	–
*Parapercis* sp.	2.3	–	–
*Parapercis xanthozona*	0.5	–	–
*Paraplotosus butleri*	0.5	–	–
*Parupeneus barberinus*	3.3	0.9	–
*Parupeneus heptacanthus*	8.8	5.2	–
*Parupeneus indicus*	16.3	15.5	–
*Parupeneus pleurostigma*	–	0.9	–
*Parupeneus* sp.	0.9	1.7	–
*Parupeneus spilurus*	–	0.9	–
*Pentapodus emeryii*	6.0	1.7	–
*Pentapodus porosus*	4.7	6.0	–
*Pentapodus* sp.	34.9	17.2	3.8
*Pentapodus vitta*	4.7	–	1.0
*Pinguipedidae* sp.	3.3	3.4	–
*Plagiotremus* sp.	0.5	–	–
*Platax batavianus*	7.4	–	1.0
*Platax orbicularis*	0.5	–	–
*Plectorhinchus caeruleonothus*	–	0.9	–
*Plectorhinchus flavomaculatus*	0.5	2.6	–
*Plectorhinchus gibbosus*	7.0	6.0	–
*Plectorhinchus vittatus*	0.5	–	–
*Plectropomus areolatus*	0.9	–	–
*Plectropomus maculatus*	18.1	16.4	1.0
*Plectropomus* sp.	19.1	11.2	–
*Polycheata* sp.	1.9	–	8.7
*Pomacanthidae* sp.	–	0.9	–
*Pomacanthus imperator*	3.7	9.5	–
*Pomacanthus semicirculatus*	–	5.2	–
*Pomacanthus sexstriatus*	7.9	4.3	–
*Pomacanthus* sp.	0.9	–	–
*Pomacentridae* sp.	9.8	3.4	4.8
*Pomacentrus nagasakiensis*	0.9	–	–
*Pristipomoides multidens*	–	–	1.9
*Pseudobalistes fuscus*	2.8	2.6	–
*Pseudobalistes* sp.	–	0.9	–
*Pseudochromis* sp.	1.9	2.6	–
*Pseudomonacanthus peroni*	–	0.9	–
*Ptereleotris* sp.	0.5	–	–
*Pterocaesio* sp.	–	0.9	–
*Pterois volitans*	–	0.9	–
*Rachycentron canadum*	2.8	3.4	3.8
*Rhina ancylostoma*	–	–	1.0
*Rhinidae* sp.	0.5	–	1.0
*Rhinobatidae* sp.	0.5	2.6	–
*Rhizoprionodon acutus*	7.9	31.0	52.9
*Rhynchobatus* sp.	3.7	11.2	2.9
*Rhynchostracion nasus*	–	0.9	3.8
*Sarda orientalis*	0.5	0.9	3.8
*Sarda* sp.	0.9	–	–
*Saurida* sp.	–	–	1.0
*Saurida undosquamis*	11.6	33.6	79.8
*Scaridae* sp.	13.0	9.5	1.0
*Scarus frenatus*	0.5	–	–
*Scarus ghobban*	1.9	4.3	–
*Scarus* sp.	7.0	1.7	–
*Scolopsis monogramma*	32.6	15.5	–
*Scolopsis* sp.	–	2.6	–
*Scolopsis taenioptera*	–	0.9	–
*Scomberoides commersonnianus*	1.9	–	1.0
*Scomberoides lysan*	1.4	–	–
*Scomberoides* sp.	3.7	0.9	–
*Scomberomorus commerson*	10.2	16.4	8.7
*Scomberomorus* sp.	9.3	12.9	3.8
*Scombridae* sp.	3.7	1.7	1.9
*Scyphozoa* sp.	0.5	–	–
*Selar* sp.	–	–	1.0
*Sepia smithi*	0.5	–	–
*Sepia* sp.	0.5	0.9	2.9
*Seriola dumerili*	0.5	–	‐
*Seriola rivoliana*	–	–	1.9
*Seriolina nigrofasciata*	2.8	19.8	38.5
*Serranidae* sp.	2.3	–	1.0
*Siganus punctatus*	0.5	2.6	–
*Siganus* sp.	0.5	7.8	–
*Sphyraena barracuda*	11.6	0.9	1.9
*Sphyraena jello*	10.7	–	–
*Sphyraena* sp.	4.2	0.9	–
*Sphyrna mokarran*	1.9	0.9	2.9
*Stegostoma tigrinum*	3.3	0.9	1.9
*Suezichthys cyanolaemus*	11.2	3.4	4.8
*Suezichthys devisi*	0.5	–	–
*Suezichthys soelae*	0.9	0.9	–
*Suezichthys* sp.	0.5	–	–
*Sufflamen fraenatum*	32.6	39.7	1.0
*Symphorus nematophorus*	38.1	14.7	–
*Synodontidae* sp.	0.9	–	2.9
*Synodus* sp.	1.9	5.2	–
*Synodus variegatus*	0.5	–	–
*Taeniurops meyeni*	1.4	–	–
*Tetraodontidae* sp.	–	0.9	–
*Teuthida* sp.	–	–	1.0
*Triglidae* sp.	–	–	1.0
*Upeneus australiae*	0.5	–	–
*Upeneus luzonius*	0.9	–	1.0
*Valenciennea* sp.	0.9	0.9	–
*Zabidius novemaculeatus*	–	0.9	–

## APPENDIX 5

Prevalence (%) of pelagic species recorded at WN, CR, and CS. Prevalence refers to the number of deployments on which a taxon was observed, out of the total number of deployments at that site

**Table:**

Binomial	WN	CR	CS
*Ablennes hians*	–	–	5
*Acanthocybium solandri*	5.8	3	18
*Alepes apercna*	3.8	3	–
*Alepes kleinii*	1.9	–	–
*Alepes* sp.	19	31	–
*Alepes vari*	1.9	–	–
*Aluterus monoceros*	27	28	18
*Aluterus scriptus*	65	47	41
*Aluterus* sp.	7.7	–	32
*Apogonidae* sp.	1.9	3	–
*Atule mate*	40	41	55
*Auxis thazard*	–	–	5
*Balaenoptera acutorostrata*	1.9	3	–
*Brachyura* sp.	1.9	–	–
*Cantherhines dumerilii*	9.6	6	5
*Cantherhines pardalis*	1.9	–	–
*Carangidae* sp.	37	44	50
*Carangoides armatus*	12	6	36
*Carangoides ferdau*	–	–	5
*Carangoides fulvoguttatus*	3.8	–	–
*Carangoides gymnostethus*	3.8	–	5
*Carangoides* sp.	9.6	3	27
*Caranx ignobilis*	1.9	–	–
*Caranx sexfasciatus*	1.9	6	–
*Caranx* sp.	3.8	–	–
*Carcharhinidae* sp.	1.9	–	–
*Carcharhinus amblyrhynchos*	21	–	–
*Carcharhinus amboinensis*	9.6	28	50
*Carcharhinus brevipinna*	3.8	6	–
*Carcharhinus falciformis*	15	3	50
*Carcharhinus galapagensis*	1.9	–	–
*Carcharhinus leucas*	1.9	6	14
*Carcharhinus limbatus*	21	6	–
*Carcharhinus obscurus*	40	28	36
*Carcharhinus plumbeus*	54	47	50
*Carcharhinus sorrah*	13	6	23
*Carcharhinus* sp.	58	53	59
*Carcharhinus tilstoni*	1.9	–	–
*Cestum veneris*	9.6	–	18
*Cheloniidae* sp.	5.8	–	5
*Clupeidae* sp.	25	13	41
*Coryphaena equiselis*	–	–	5
*Coryphaena hippurus*	1.9	3	5
*Decapterus macarellus*	–	–	5
*Decapterus* sp.	69	66	86
*Delphinidae* sp.	1.9	–	–
*Disteira major*	1.9	–	–
*Echeneidae* sp.	–	3	–
*Echeneis naucrates*	48	63	86
*Elagatis bipinnulata*	15	–	–
*Elapidae* sp.	15	9	5
*Ephippidae* sp.	–	6	–
*Eubalichthys caeruleoguttatus*	3.8	6	9
*Fistularia* sp.	9.6	6	18
*Galeocerdo cuvier*	5.8	9	5
*Gnathanodon speciosus*	5.8	3	5
*Hydrophis ocellatus*	1.9	–	5
*Hydrophis* sp.	1.9	3	9
*Hydrozoa* sp.	37	13	18
*Istiompax indica*	3.8	3	18
*Istiophoridae* sp.	–	3	–
*Istiophorus platypterus*	7.7	3	–
*Juvenile* sp.	37	13	18
*Katsuwonus pelamis*	1.9	–	–
*Labroides dimidiatus*	–	–	5
*Lagocephalus sceleratus*	1.9	–	–
*Makaira nigricans*	3.8	3	23
*Megalaspis cordyla*	1.9	–	–
*Metavelifer multiradiatus*	–	–	5
*Mobula kuhlii*	–	–	5
*Mobula* sp.	3.8	6	5
*Monacanthidae* sp.	27	6	14
*Natator depressa*	1.9	–	–
*Naucrates ductor*	–	–	5
*Nomeidae* sp.	1.9	–	5
*Platax* sp.	–	3	9
*Platax teira*	1.9	–	–
*Priacanthus* sp.	1.9	–	9
*Psenes* sp.	3.8	6	32
*Rachycentron canadum*	1.9	6	32
*Remora remora*	–	3	5
*Remora* sp.	–	–	5
*Sardinella* sp.	1.9	–	–
*Scomberoides lysan*	3.8	–	–
*Scomberomorus commerson*	23	–	–
*Scomberomorus* sp.	9.6	–	5
*Scombridae* sp.	3.8	6	–
*Scyphozoa* sp.	42	25	27
*Selar boops*	1.9	–	–
*Selar* sp.	1.9	3	–
*Sepia* sp.	1.9	–	–
*Seriola* sp.	17	31	–
*Seriolina nigrofasciata*	31	38	68
*Sphyraena barracuda*	60	6	23
*Sphyraena* sp.	–	–	5
*Sphyrna mokarran*	1.9	16	18
*Teuthida* sp.	–	3	–
